# Metabolic engineering strategies for sustainable de novo isobutylamine production by *Corynebacterium glutamicum*

**DOI:** 10.1186/s13068-026-02831-w

**Published:** 2026-10-11

**Authors:** Tim Prasun, Susanne Müller, Fynn Stegelmann, Bastian Blombach, Volker F. Wendisch

**Affiliations:** 1 Genetics of Prokaryotes, Faculty of Biology and Center for Biotechnology (CeBiTec), Bielefeld University, Bielefeld, Germany; 2 Microbial Biotechnology, Campus Straubing for Biotechnology and Sustainability, Technical University of Munich, Straubing, Germany; 3 SynBiofoundry@TUM, Technical University of Munich, Straubing, Germany

**Keywords:** *Corynebacterium glutamicum*, Metabolic engineering, Isobutylamine overproduction, Short-chain primary amine, Alkylamine

## Abstract

**Background:**

Short-chain primary amines are a versatile group of molecules in biological systems, derived from the decarboxylation of amino acids or the transamination of carbonyl-containing molecules. Isobutylamine, the decarboxylation product of l-valine, has applications in chemical synthesis of pharmaceuticals, herbicides, fungicides and specialized chemicals, where it is especially interesting as a substitute for petrochemical compounds. Since the chemical synthesis of short-chain primary amines is hazardous and environmentally unsustainable, we established an efficient biosynthetic approach in this work.

**Results:**

The natural amino acid producer *Corynebacterium glutamicum* is an ideal chassis for the production of amino acid-derived molecules. In this study, we assessed the toxicity of several relevant short-chain primary amines on *C. glutamicum*. A crude extract assay demonstrated the decarboxylation of l-valine to isobutylamine by the l-valine decarboxylase VlmD_Sv_ from *Streptomyces viridifaciens*. This enzyme was instrumental to extend production of pyruvate in three engineered strains to yield l-valine and subsequently isobutylamine. To ensure intracellular l-valine supply, the extracellular l-valine accumulation was decreased up to 20-fold by deletion of the genes for the branched-chain amino acid exporter BrnFE_Cg_ and/or overexpression of the l-valine importer gene *brnQ*_*Cg*_. Surprisingly, expression of the transhydrogenase genes *pntAB*_*Ec*_ from *Escherichia coli* did not improve but drastically reduced isobutylamine production. Biochemical analysis revealed that *pntAB*_*Ec*_ expression depolarized the membrane potential.

**Conclusions:**

This study demonstrated the highest production of isobutylamine and the highest production of any microbial short-chain primary amine in minimal medium. In shake flask cultivation, an isobutylamine titer of 12.1 g/L and a yield of 0.74 mol isobutylamine/mol glucose were obtained. Transfer to a 3 L fed-batch bioreactor fermentation was successful and a titer of 33.2 g/L was reached within 64 h with a yield of 0.47 mol/mol and a volumetric productivity of 0.52 g/l/h. Moreover, isobutylamine production on a second-generation feedstock was shown, highlighting the potential of sustainable bio-based production of isobutylamine.

**Supplementary Information:**

The online version contains supplementary material available at https://doi.org/10.1186/s13068-026-02831-w.

## Introduction

The production of bulk chemicals remains heavily dependent on finite petrochemical feedstocks, which inherently pose an obstacle to achieving a sustainable and circular economy. Aliphatic amines constitute a highly important class of compounds for various chemical industries with a market of USD 3.8 billion in 2024 and projected to reach USD 5.7 billion by 2033 [39]. Notably, more than 40% of small molecule pharmaceuticals can be classified as amines [16, 35]. Furthermore, they are prevalent precursors in agrochemistry for insecticide, herbicide and fungicide synthesis [43]. The high reactivity of polyvalent amines is frequently exploited to condensate polymers, a major example being nylon synthesis with bioproduced putrescine [36, 41, 42].

The manufacture of aliphatic amines is predominantly achieved by reductive amination of fossil-derived carbonyls. Harsh reaction conditions, unsafe reactants and energy-intensive processes substantiate bio-based productions. Advances in metabolic engineering and systems biology opened the doors for rational pathway design for the targeted production of desired compounds from renewable carbon sources that do not have competing uses in food and feed. Short-chain primary amines (SCPAs) are amines with an aryl or alkyl rest with a chain length of 1–7 carbons [24]. They are particularly attractive targets for in vivo production, since they often derive from enzymatic transamination of an aldehyde or ketone or decarboxylation of an amino acid [14, 33]. For example, the four-carbon isobutylamine arises from decarboxylation of l-valine in *Streptomyces viridifaciens* during the biosynthesis of the antibiotic agent valinomycin [1]. In industry, isobutylamine is extensively applied as corrosion inhibitor, rubber processing chemical, antiknock agent and as precursor for the synthesis of structurally complex compounds owing to its reactive primary amine functionality [43].

In this study, we report the highest microbial production titer for any SCPA in minimal medium (Table 1). We assessed the toxicity of different SCPAs for the wild-type *Corynebacterium glutamicum* strain ATCC 13032 and found it able to tolerate high concentrations of isobutylamine. The availability of the precursor l-valine was increased by overexpression of the l-valine biosynthetic genes in pyruvate-producing strains. By subsequent decarboxylation of l-valine, significant titers of isobutylamine were reached. Second-generation feedstocks could be used for isobutylamine production and fermentation in 3 L bioreactors increased the titer, underlining the potential of biotechnological sustainable processes for isobutylamine production in the context of circular economy.

**Table 1:** Growth rates, isobutylamine titers after 72 h obtained with various A16-P-derived isobutylamine producer strains with and without overexpression of *pntAB*_*Ec*_

Strain	Growth rate µ [h^−1^]	Isobutylamine [mM]
A16-P (pJC4*ilvBNCD*_*Cg*_) (pECXT99a–*vlmD*_*Sv*_)	0.10 ± 0.01	109.2 ± 9.3
A16-P (pJC4*ilvBNCD*_*Cg*_–***pntAB***_***Ec***_) (pECXT99a–*vlmD*_*Sv*_)	0.04 ± 0.00	4 ± 0.2
A16-P (pJC4*ilvBNCD*_*Cg*_) (pECXT99a–*vlmD*_*Sv*_–*brnQ*_*Cg*_)	0.13 ± 0.01	163.5 ± 24
A16-P (pJC4*ilvBNCD*_*Cg*_–***pntAB***_***Ec***_) (pECXT99a–*vlmD*_*Sv*_–*brnQ*_*Cg*_)	0.06 ± 0.01	3.8 ± 0.1
A16-P Δ*brnFE* (pJC4*ilvBNCD*_*Cg*_) (pECXT99a–*vlmD*_*Sv*_)	0.13 ± 0.01	163.4 ± 6.3
A16-P Δ*brnFE* (pJC4*ilvBNCD*_*Cg*_–***pntAB***_***Ec***_) (pECXT99a–*vlmD*_*Sv*_)	0.02 ± 0.01	3.1 ± 0.7
A16-P Δ*brnFE* (pJC4*ilvBNCD*_*Cg*_) (pECXT99a–*vlmD*_*Sv*_–*brnQ*_*Cg*_)	0.13 ± 0.01	165.7 ± 4.0
A16-P Δ*brnFE* (pJC4*ilvBNCD*_*Cg*_–***pntAB***_***Ec***_) (pECXT99a–*vlmD*_*Sv*_–*brnQ*_*Cg*_)	0.03 ± 0.01	2.6 ± 0.3

**Table 2:** Relevant plasmids used in this study

Plasmid	Relevant characteristics	Antibiotic resistance	References
pRG_Duet3	Dual inducible *C. glutamicum*/*E. coli* shuttle vector. *P*_*tac*_ (IPTG), *lacI*^*Q*^, *P*_*tetR*/*tetA*_ (ATc), tetR, pBL1 OriV_*Cg*_, colE1 OriV_*Ec*_	Cm^R^	This work
pRG_Duet3–*vlmD*_*Sv*_	pRG_Duet3 carrying codon harmonized *vlmD*_*Sv*_ from *S. viridifaciens* under control of *P*_*tetR*/*tetA*_	Cm^R^	This work
pRG_Duet3–*vlmD*_*Sv*_–*ilvE*_*Cg*_	pRG_Duet3–*vlmD*_*Sv*_ carrying *ilvE*_*Cg*_ from *C. glutamicum* under control of *P*_*tac*_	Cm^R^	This work
pJC4	*C. glutamicum*/*E. coli* shuttle vector. *P*_*tac*_ (IPTG), *lacI*^*Q*^, pCG1 OriV_*Cg*_, p15A OriV_*Ec*_	Kan^R^	[11]
pJC4*ilvBNCD*_*Cg*_	pJC4 carrying *ilvBNCD*_*Cg*_ from *C. glutamicum* in an artificial operon under control of *P*_*tac*_	Kan^R^	[38]
pJC4*ilvBNCD*_*Cg*_–*pntAB*_*Ec*_	pJC4*ilvBNCD*_*Cg*_ carrying *pntAB*_*Ec*_ from *E. coli* in an artificial operon with *ilvBNCD*_*Cg*_	Kan^R^	[5]
pJC4*ilvBNCD*_*Cg*_–*leuA*_*Cg*_^G462D^	pJC4*ilvBNCD*_*Cg*_ carrying feedback resistant *leuA*_*Cg*_^G462D^ from *C. glutamicum* in an artificial operon with *ilvBNCD*_*Cg*_	Kan^R^	[9]
pECXT99A	*C. glutamicum*/*E. coli* shuttle vector. *P*_*trc*_ (IPTG), *lacI*^q^, pGA1 oriV_*Cg*_, pMB1 oriV_*Ec*_	Tet^R^	[25]
pECXT99A–*vlmD*_*Sv*_	pECXT99A carrying codon harmonized *vlmD*_*Sv*_ under control of *P*_*trc*_	Tet^R^	This work
pECXT99A–*vlmD*_*Sv*_–*brnQ*_*Cg*_	pECXT99A–*vlmD*_*Sv*_ carrying *brnQ*_*Cg*_ in an artificial operon with *vlmD*_*Sv*_	Tet^R^	This work
pEKEx3–*xylA*_*Xc*_*B*_*Cg*_	*C. glutamicum*/*E. coli* shuttle vector pEKEx3 derivative carrying *xylA*_*Xc*_ from *Xanthomonas campestris* and *xylB*_*Cg*_ from *C. glutamicum*. *P*_*tac*_ (IPTG), *lacI*^q^, pBL1 oriV_*Cg*_, pUC18 oriV_*Ec*_	Spec^R^	[30]
pK19*mobsacB*	Suicide vector for conjugations ColE1 oriV_*Ec*_, oriT, *mob* and *sacB* gene from *Bacillus subtilis*	Km^R^	[40]
pK19*mobsacB*–∆*brnFE*	pK19*mobsacB* carrying truncated *brnFE* genes from *C. glutamicum*	Km^R^	This work

## Materials and methods

### Bacterial strains and growth conditions

The plasmids and bacterial strains used in this study are listed in Tables 2 and 3. *E coli* DH5α was used for plasmid construction and storage, *E. coli* S17-1 was used as donor strain during conjugations for gene deletions and *E. coli* BL21(DE3) as host for protein expression [18, 40, 45, 47]. *E. coli* and *C. glutamicum* over-night precultures were inoculated from Lysogeny broth (LB) plates into LB medium in 10 mL volume in 100 mL baffled shake flasks [4]. Precultures were incubated in 37 °C and 180 rpm for *E. coli* and 30 °C and 120 rpm for *C. glutamicum*.

**Table 3:** Relevant strains used in this study

Strain	Relevant characteristics	References
*C. glutamicum* WT	wild type, ATCC 13032	ATCC
ISO-P	*C. glutamicum* WT with deletions Δ*aceE* Δ*pqo* Δ*ilvE* Δ*ldhA Δmdh*	[5]
ELB-P	*C. glutamicum* WT with deletions *ΔaceE Δpqo ΔldhA ΔC–T ilvN ΔalaT ΔavtA*	[53]
A16-P	*C. glutamicum* WT with promotor replacement Δ*P*_*aceE*_::*P*_*dap-A16*_ and deletions Δ*pqo* Δ*ppc*	[8]
ISO-P Δ*brnFE*	ISO-P with deletion Δ*brnFE*	This work
ELB-P Δ*brnFE*	ELB-P with deletion Δ*brnFE*	This work
A16-P Δ*brnFE*	A16-P with deletion Δ*brnFE*	This work
ISO-P (pJC4*ilvBNCD*_*Cg*_–*pntAB*_*Ec*_) (pRG_Duet3–*vlmD*_*Sv*_–*ilvE*_*Cg*_)	ISO-P harboring pJC4*ilvBNCD*_*Cg*_–*pntAB*_*Ec*_ and pRG_Duet3–*vlmD*_*Sv*_–*ilvE*_*Cg*_	This work
ISO-P Δ*brnFE* (pJC4*ilvBNCD*_*Cg*_–*pntAB*_*Ec*_) (pRG_Duet3–*vlmD*_*Sv*_–*ilvE*_*Cg*_)	ISO-P Δ*brnFE* harboring pJC4*ilvBNCD*_*Cg*_–*pntAB*_*Ec*_ and pRG_Duet3–*vlmD*_*Sv*_–*ilvE*_*Cg*_	This work
ELB-P (pJC4*ilvBNCD*_*Cg*_–*pntAB*_*Ec*_) (pRG_Duet3–*vlmD*_*Sv*_–*ilvE*_*Cg*_)	ELB-P harboring pJC4*ilvBNCD*_*Cg*_–*pntAB*_*Ec*_ and pRG_Duet3–*vlmD*_*Sv*_–*ilvE*_*Cg*_	This work
ELB-P Δ*brnFE* (pJC4*ilvBNCD*_*Cg*_–*pntAB*_*Ec*_) (pRG_Duet3–*vlmD*_*Sv*_–*ilvE*_*Cg*_)	ELB-P Δ*brnFE* harboring pJC4*ilvBNCD*_*Cg*_–*pntAB*_*Ec*_ and pRG_Duet3–*vlmD*_*Sv*_–*ilvE*_*Cg*_	This work
A16-P (pJC4*ilvBNCD*_*Cg*_–*pntAB*_*Ec*_) (pRG_Duet3–*vlmD*_*Sv*_–*ilvE*_*Cg*_)	A16-P harboring pJC4*ilvBNCD*_*Cg*_–*pntAB*_*Ec*_ and pRG_Duet3–*vlmD*_*Sv*_–*ilvE*_*Cg*_	This work
A16-P Δ*brnFE* (pJC4*ilvBNCD*_*Cg*_–*pntAB*_*Ec*_) (pRG_Duet3–*vlmD*_*Sv*_–*ilvE*_*Cg*_)	A16-P Δ*brnFE* harboring pJC4*ilvBNCD*_*Cg*_–*pntAB*_*Ec*_ and pRG_Duet3–*vlmD*_*Sv*_–*ilvE*_*Cg*_	This work
A16-P (pJC4*ilvBNCD*_*Cg*_–*pntAB*_*Ec*_) (pECXT99A–*vlmD*_*Sv*_)	A16-P harboring pJC4*ilvBNCD*_*Cg*_–*pntAB*_*Ec*_ and pECXT99A–*vlmD*_*Sv*_	This work
A16-P (pJC4*ilvBNCD*_*Cg*_–*pntAB*_*Ec*_) (pECXT99A–*vlmD*_*Sv*_–*brnQ*_*Cg*_)	A16-P harboring pJC4*ilvBNCD*_*Cg*_–*pntAB*_*Ec*_ and pECXT99A–*vlmD*_*Sv*_–*brnQ*_*Cg*_	This work
A16-P Δ*brnFE* (pJC4*ilvBNCD*_*Cg*_–*pntAB*_*Ec*_) (pECXT99A–*vlmD*_*Sv*_)	A16-P Δ*brnFE* harboring pJC4*ilvBNCD*_*Cg*_–*pntAB*_*Ec*_ and pECXT99A–*vlmD*_*Sv*_	This work
A16-P Δ*brnFE* (pJC4*ilvBNCD*_*Cg*_–*pntAB*_*Ec*_) (pECXT99A–*vlmD*_*Sv*_–*brnQ*_*Cg*_)	A16-P Δ*brnFE* harboring pJC4*ilvBNCD*_*Cg*_–*pntAB*_*Ec*_ and pECXT99A–*vlmD*_*Sv*_–*brnQ*_*Cg*_	This work
A16-P (pJC4*ilvBNCD*_*Cg*_) (pECXT99A–*vlmD*_*Sv*_)	A16-P harboring pJC4*ilvBNCD*_*Cg*_ and pECXT99A–*vlmD*_*Sv*_	This work
A16-P (pJC4*ilvBNCD*_*Cg*_) (pECXT99A–*vlmD*_*Sv*_–*brnQ*_*Cg*_)	A16-P harboring pJC4*ilvBNCD*_*Cg*_ and pECXT99A–*vlmD*_*Sv*_–*brnQ*_*Cg*_	This work
A16-P Δ*brnFE* (pJC4*ilvBNCD*_*Cg*_) (pECXT99A–*vlmD*_*Sv*_)	A16-P Δ*brnFE* harboring pJC4*ilvBNCD*_*Cg*_ and pECXT99A–*vlmD*_*Sv*_	This work
A16-P Δ*brnFE* (pJC4*ilvBNCD*_*Cg*_) (pECXT99A–*vlmD*_*Sv*_–*brnQ*_*Cg*_)	A16-P Δ*brnFE* harboring pJC4*ilvBNCD*_*Cg*_ and pECXT99A–*vlmD*_*Sv*_–*brnQ*_*Cg*_	This work
A16-P (pJC4) (pECXT99A–*vlmD*_*Sv*_)	A16-P harboring pJC4 and pECXT99A–*vlmD*_*Sv*_	This work
A16-P (pJC4) (pECXT99A–*vlmD*_*Sv*_–*brnQ*_*Cg*_)	A16-P harboring pJC4 and pECXT99A–*vlmD*_*Sv*_–*brnQ*_*Cg*_	This work
A16-P Δ*brnFE* (pJC4) (pECXT99A–*vlmD*_*Sv*_)	A16-P Δ*brnFE* harboring pJC4 and pECXT99A–*vlmD*_*Sv*_	This work
A16-P Δ*brnFE* (pJC4) (pECXT99A–*vlmD*_*Sv*_–*brnQ*_*Cg*_)	A16-P Δ*brnFE* harboring pJC4 and pECXT99A–*vlmD*_*Sv*_–*brnQ*_*Cg*_	This work
A16-P (pJC4*ilvBNCD*_*Cg*_–*leuA*_*Cg*_^G462D^) (pECXT99A–*vlmD*_*Sv*_)	A16-P harboring pJC4*ilvBNCD*_*Cg*_–*leuA*_*Cg*_^G462D^ and pECXT99A–*vlmD*_*Sv*_	This work
A16-P (pJC4*ilvBNCD*_*Cg*_–*leuA*_*Cg*_^G462D^) (pECXT99A–*vlmD*_*Sv*_–*brnQ*_*Cg*_)	A16-P harboring pJC4*ilvBNCD*_*Cg*_–*leuA*_*Cg*_^G462D^ and pECXT99A–*vlmD*_*Sv*_–*brnQ*_*Cg*_	This work
A16-P Δ*brnFE* (pJC4*ilvBNCD*_*Cg*_–*leuA*_*Cg*_^G462D^) (pECXT99A–*vlmD*_*Sv*_)	A16-P Δ*brnFE* harboring pJC4*ilvBNCD*_*Cg*_–*leuA*_*Cg*_^G462D^ and pECXT99A–*vlmD*_*Sv*_	This work
A16-P Δ*brnFE* (pJC4*ilvBNCD*_*Cg*_–*leuA*_*Cg*_^G462D^) (pECXT99A–*vlmD*_*Sv*_–*brnQ*_*Cg*_)	A16-P Δ*brnFE* harboring pJC4*ilvBNCD*_*Cg*_–*leuA*_*Cg*_^G462D^ and pECXT99A–*vlmD*_*Sv*_–*brnQ*_*Cg*_	This work
A16-P Δ*brnFE* (pJC4*ilvBNCD*_*Cg*_) (pECXT99A–*vlmD*_*Sv*_) (pEKEx3–*xylA*_*Xc*_*B*_*Cg*_)	A16-P Δ*brnFE* (pJC4*ilvBNCD*_*Cg*_) (pECXT99A–*vlmD*_*Sv*_) harboring pEKEx3–*xylA*_*Xc*_*B*_*Cg*_	This work
A16-P Δ*brnFE* (pJC4) (pECXT99A–*vlmD*_*Sv*_) (pEKEx3–*xylA*_*Xc*_*B*_*Cg*_)	A16-P Δ*brnFE* (pJC4) (pECXT99A–*vlmD*_*Sv*_) harboring pEKEx3–*xylA*_*Xc*_*B*_*Cg*_	This work
A16-P Δ*brnFE* (pJC4*ilvBNCD*_*Cg*_) (pECXT99A–*vlmD*_*Sv*_–*brnQ*_*Cg*_) (pEKEx3–*xylA*_*Xc*_*B*_*Cg*_)	A16-P Δ*brnFE* (pJC4*ilvBNCD*_*Cg*_) (pECXT99A–*vlmD*_*Sv*_–*brnQ*_*Cg*_) harboring pEKEx3–*xylA*_*Xc*_*B*_*Cg*_	This work
A16-P Δ*brnFE* (pJC4) (pECXT99A–*vlmD*_*Sv*_–*brnQ*_*Cg*_) (pEKEx3–*xylA*_*Xc*_*lB*_*Cg*_)	A16-P Δ*brnFE* (pJC4) (pECXT99A–*vlmD*_*Sv*_–*brnQ*_*Cg*_) harboring pEKEx3–*xylA*_*Xc*_*B*_*Cg*_	This work

*C. glutamicum* main cultures were washed twice in TN buffer pH 6.3 (10 mM Tris–HCl, 150 mM NaCl) and inoculated in 10 mL CGXII minimal medium supplied with 0.195 mM protocatechuic acid (PCA) and 0.82 µM biotin to an OD_600_ of 1 and incubated under the same conditions as the precultures [31]. OD_600_ was measured with a V-1200 spectrophotometer (VWR, Radnor, PA, United States). ISO-P-derived strains were supplied with 2 mM l-leucine, l-valine and l-isoleucine; ELB-P-derived strains were supplied with 2 mM l-alanine to compensate their respective auxotrophies [5, 53]. All *C. glutamicum* strains were cultivated with 4% glucose and 1% K-acetate when compared directly with each other. When A16-P-derived strains were cultivated separately, 4% glucose was used as the sole carbon source, without the addition of acetate. 1 mM Isopropyl β-D-1-thiogalactopyranoside (IPTG) and when pRG_Duet3-derived plasmids were used, 3 µM anhydrotetracycline (ATc) were additionally added to induce the respective promoters. Where necessary, kanamycin (25 µg/mL), chloramphenicol (6 µg/mL for *C. glutamicum*, 30 µg/mL for *E. coli*) and tetracycline (5 µg/mL) were added.

### Toxicity assessment for SCPAs

Toxicity tests were conducted with the *C. glutamicum* WT (ATCC 13032) in 3 mL CGXII with 4% glucose in Duetz deep-well microtiter plates with an initial inoculum to an OD_600_ of 1 at 220 rpm and 30 °C [12]. Covers with 2.5 mm holes were used for increased oxygen supplementation. All amines were bought as HCl salt, the pH of the solution was set to 7.0. Increasing concentrations between 0 and 500 mM were added to the medium.

### Fed-batch cultivation

Fed-batch cultivation was carried out in a 3.7 L KLF bioreactor (Bioengineering AG, Wald, Switzerland) with three Rushton turbines in an initial volume of 2 L CGXII with kanamycin (25 µg/mL) and tetracycline (5 µg/mL). The fermenter was inoculated with the strain *C. glutamicum* A16-P Δ*brnFE* (pJC4*ilvBNCD*_*Cg*_) (pECXT99a–*vlmD*_*Sv*_–*brnQ*_*Cg*_) to an initial OD_600_ of 0.5 from a CGXII preculture that had been inoculated from an initial LB preculture. During cultivation, the temperature was kept constant at 30 °C and the pH was kept at 7 by influx of 100 g/L H_2_PO_4_ or 250 g/L NH_4_^+^. A headspace overpressure of 1.4 bar was held throughout the cultivation. The feed consisted of 400 g/L glucose supplied with PCA (0.195 mM), Biotin (0.82 µM), CGXII-trace elements and IPTG (1 mM). The feed was added when the relative dissolved oxygen rate (rDOS) surpassed 60%, indicating low glucose concentrations, and was stopped when it decreased below 30%. The agitator speed was initially set to 350 rpm and then gradually increased with increasing biomass depending on rDOS. Samples for HPLC analysis and OD_600_ measurements were taken by an automatic sampler every 2 h for the first 48 h and then every 4 h and stored at 4 °C. Initial airflow was set to 1.25 NL min^−1^, the off-gas was passed through a gas wash bottle filled with 3 M HCl to catch any residual isobutylamine. Antifoam 204 was added manually to control foam formation.

### Recombinant DNA work and strain construction

Chemo-competent *E. coli* were prepared with the CaCl_2_ method and transformed using heat shock [29]. Electro-competent *C. glutamicum* were prepared accordingly and transformed by electroporation at 2.5 kV, 200 Ω, and 25 μF [13]. Plasmids were constructed utilizing Gibson Assembly [17]. DNA fragments were amplified using either Phusion High-Fidelity DNA polymerase or ALLin™ HiFi DNA Polymerase according to the manufacturer’s protocol with primers given in Table 4 (New England Biolabs, United Kingdom or HighQu GmbH, Germany). *C. glutamicum* genes were amplified from the WT genome. The *vlmD*_*Sv*_ gene was codon harmonized [19] and ordered as gene synthesis (Twist Bioscience, South San Francisco, USA). An optimized RBS was added for all newly cloned genes in this study using the RBS Calculator v.2.1 (De Novo DNA, Salis Lab). For compatibility with pJC4-derived plasmids, the dual inducible vector pRG_Duet3 was constructed by exchanging the Kan^R^ resistance gene from pRG_Duet2 with a Cm^R^ resistance gene. pRG_Duet3 was cut with either *Bam*HI for the IPTG-inducible MCS or *Nhe*I for the ATc-inducible MCS; pECXT99A and pK19*mobsacB* were cut with *Xba*I for Gibson assembly. Genomic deletions were performed utilizing conjugation of pK19*mobsacB*–Δ*brnFE* from *E. coli* S17-1 into *C. glutamicum* strains with subsequent homologous recombination [40]. Deletions and constructed plasmids were verified by colony PCR and sequencing.

**Table 4:** Relevant primers used in this study

Primer	Sequence	Description
RBS_ilvE_fw	GGCACAACACGCAGCCTGAAGTTGGCTTAAAAAGGAGAGTATTTTATGACGTCATTAGAGTTCACAGTAACCC	Forward primer for *ilvE*_*Cg*_ amplification, adds optimized RBS
ilvE_pRG_Duet3_Fw	CCTGCAGGTCGACTCTAGAGGGCACAACACGCAGCC	Addition of forward overhang for pRG_Duet3
ilvE_pRG_Duet3_Rv	GAGCTCGGTACCCGGGGATCTTAGCCAACCAGTGGGTAAAGCCATC	Addition of reverse overhang for pRG_Duet3
pRG_Duet2_Lin1	AACACCCCTTGTATTACTGTTTATGTAAGCA	Primer for linearization of pRG_Duet2 without Kan^R^
pRG_Duet2_Lin2	TCAGAATTGGTTAATTGGTTGTAACACTGG	Primer for linearization of pRG_Duet2 without Kan^R^
Cm_res_Fw	ACAGTAATACAAGGGGTGTTGATATCTGGCGAAAATGAGACGTTGATC	Addition of forward overhang for pRG_Duet2 onto Cm^R^ from pRG_Duet1
Cm_res_Rv	AACCAATTAACCAATTCTGAAACACTAGTGCTTGGATTCTCACCA	Addition of reverse overhang for pRG_Duet2 onto CmR from pRG_Duet1
vlmD _pRG_Duet3_Fw	AGAGGAGGAAAGGGTATACGGGCATTACCCTTACAAAAGGGCATT	Forward primer for *vlmD*_*Sv*_ amplification for pRG_Duet3*NheI
vlmD _pRG_Duet3_Rv	GTACAATTTAAATCCTAGGGTTAGGATCCGCCGCCATCT	Reverse primer for *vlmD*_*Sv*_ amplification for pRG_Duet3*NheI
pRG_Duet3_IPTG_Fw	CGCCGACATCATAACGGTTC	Forward sequencing primer for pRG_Duet3 (IPTG-inducible MCS) and pECXT99A
pRG_Duet3_IPTG_Rv	ACTTCTGAGTTCGGCATGGG	Reverse sequencing primer for pRG_Duet3 (IPTG-inducible MCS) and pECXT99A
pRG_Duet3_ATc_Fw	CTGGGCGAGTTTACGGGTTG	Forward sequencing primer for pRG_Duet3 (ATc-inducible MCS)
pRG_Duet3_ATc_Rv	CACGTCATTTACCGGGTCAC	Reverse sequencing primer for pRG_Duet3 (ATc-inducible MCS)
A_brnFE_pK19	TGCATGCCTGCAGGTCGACTCGACTACTGGAACAACGTTGATGAG	Amplification of ∆*brnFE* fragment from deletion strain
D_brnFE_pK19	GGTACCCGGGGATCCTCAGCTAACCAGCGGTCAAGAAAC	Amplification of ∆*brnFE* fragment from deletion strain
dbrnFE_fw_ver_seq	GCACCAGGATGATGACGACTTC	Forward verification primer of *brnFE* deletion
dbrnFE_rv_ver_seq	GACCTTGCCGATTAACAACCCAG	Reverse verification primer of *brnFE* deletion
vlmD_pECXT99A_Fw	GCTCGGTACCCGGGGATCCTGGCATTACCCTTACAAAAGGGCATT	Forward primer for *vlmD*_*Sv*_ amplification for pECXT99A*XbaI
vlmD_pECXT99A_Rv	TGCCTGCAGGTCGACTCTAGTTAGGATCCGCCGCCATCT	Reverse primer for *vlmD*_*Sv*_ amplification for pECXT99A*XbaI
vlmD_Ov_brnQ_Rv	GGAACCAGTTCCCTTGCTTGTTAGGATCCGCCGCCATCT	Reverse primer for *vlmD*_*Sv*_ amplification, adds overhang for *brnQ*_*Cg*_
brnQ_opt_RBS_Fw	CAAGCAAGGGAACTGGTTCCCAACACCAAAAAGGAGGTTTGTTATGAGTAAAAAGTCTGTCCTGATTACTTCTTTG	Forward primer for *brnQ*_*Cg*_ amplification, adds optimized RBS
brnQ_pEXCT99A_RV	TGCCTGCAGGTCGACTCTAGTTAGACGGAAATGGATTCCTTCTTTGCA	Reverse primer for *brnQ*_*Cg*_ amplification pECXT99A*XbaI

### Crude extract assay to determine VlmD_Sv_ activity

A 50 mL M9 with CGXII-trace elements main culture of *E. coli* BL21(DE3) (pRG_Duet3–*vlmD*_*Sv*_) and the respective empty vector strain were inoculated to an OD_600_ of 1 from LB-precultures and expression of proteins was induced with 3 μM ATc. Cells were harvested at an OD_600_ of 3–5 and washed twice with and then resuspended with TN buffer pH 7.0. Cells were disrupted by sonification on ice by an UP200S Ultrasonic Processor (Hielsche Ultrasound Technology) with a 55% amplitude and 0.5 cycles for 3 min. The crude extract was harvested at 4 °C and purified from low molecular weight molecules and salts by a PD-10 desalting column according to manufacturer’s protocol and eluted in 50 mM NaH_2_PO_4_. Protein concentrations were determined using Bradford assay [7] with bovine serum albumin (BSA) as standards. The reaction buffer pH 7.0 was supplied with 1 mM PLP as coenzyme and 20 mM l-valine as substrate in a final volume of 1.5 mL [15]. The assay was performed in *C. glutamicum* like conditions at 30 °C, pH 7.0. The reaction was stopped by adding 1:1 methanol; samples for HPLC measurement were taken at 0 min, 2.5 min, 5 min, 7.5 min, 10 min, 15 min, 30 min and 1 h. The assay was done with varying protein concentrations in duplicates.

### Quantification of metabolites with HPLC analysis

High-performance liquid chromatography was used for the quantification of amines, and carbohydrates. Samples for measurements of amines were diluted by 1:100 with MilliQ water to a final volume of 1 mL and 100 μM asparagine were used as internal standard. The samples were automatically derivatized with *ortho*-phtalaldehyde (OPA) to form fluorogenic derivatives [37]. As mobile phases 0.25% Na-acetate (pH 6.0) and methanol were used. The gradients were: 0 min 20% B; 1 min 38% B; 3.5 min 46% B; 5.5 min 65% B; 7 min 70% B; 9 min 75% B; 11 min 85% B; 13 min 20% B; 15 min 20% B. Flow rates were 0.7 mL/min for 0 min and 1.2 mL/min at 1 min and after. For separation, a pre-column (LiChrospher 100 RP18 EC-5μ (40 × 4 mm), CS-Chromatographie Service GmbH) and a main column (LiChrospher 100 RP18 EC-5μ (125 × 4 mm), CS–Chromatographie Service GmbH) were used. A fluorescence light detector (FLD G1321A, 1200 series, Agilent Technologies) was used with an excitation wavelength of 263 nm and emission detection at 310 nm.

For carbohydrate quantification, a column for measurements of carbonic acids was used (Aminex 300 × 8 mm, 10 μm particle size, 25 Å pore diameter, CS–Chromatographie Service GmbH). As liquid phase 5 mM H_2_SO_4_ was used with a steady flow rate of 0.8 mL/min at 60 °C for 17 min. For detection a diode array detector (DAD, 1200 series, Agilent Technologies) was used at a wavelength of 210 nm.

### Membrane potential analysis

The carbocyanine dye 3,3′-diethyloxacarbocyanine iodide (DiOC_2_(3)) can be used to analyze the membrane potential (MP). DiOC_2_(3) monomers exhibit green fluorescence. Depending on the MP, the dye molecules self-associate, causing a shift from green to red fluorescence. The extent of self-association increases with the magnitude of the MP. As cyanine dyes are positively charged, they accumulate intracellularly in response to the negative MP gradient, resulting in increased self-association. Therefore, the red-to-green fluorescence ratio is a reliable indicator of the MP [34]. The membrane potential analysis was conducted in accordance with a previously established protocol for *C. glutamicum* [32]. Cells were grown in CGXII supplied with 4% glucose and different concentrations of isobutylamine. Culture samples were diluted to an OD_600_ of 0.05 with phosphate buffered saline (PBS) (137 mM NaCl, 2.7 mM KCl, 10 mM Na_2_HPO_4_, 1.8 mM KH_2_PO_4_, pH 7.4). If indicated, diluted samples were treated with 50 µM Carbonyl cyanide 3-chlorophenylhydrazone (CCCP) as MP-decoupler control or with isobutylamine analyzing its direct influence. The cells were then stained with 30 µM DiOC_2_(3) and incubated at room temperature for 30 min. The fluorescence was measured at the single-cell level by flow cytometry using a CytoFLEX SRT cell sorter from Beckman Coulter. DiOC_2_(3) is excited at 488 nm, and the green and red fluorescence emissions are detected using 525/40 nm and 610/20 nm bandpass filters, respectively. The data were acquired and analyzed with the CytExpert SRT software (version 1.2.0.10004, Beckman Coulter). 10 000 cell events per sample were acquired at a flow rate of 10 µL s^−1^.

### Statistical analysis

Statistical analyses were performed using OriginPro (OriginLab Corporation). Differences between groups were analyzed using one-way analysis of variance (ANOVA), followed by Tukey’s honestly significant difference (HSD) test for multiple comparisons. A *p* value < 0.05 was considered statistically significant.

## Results

### Characterization of the effects of SCPAs on growth of *C. glutamicum* WT

**Fig. 1:**
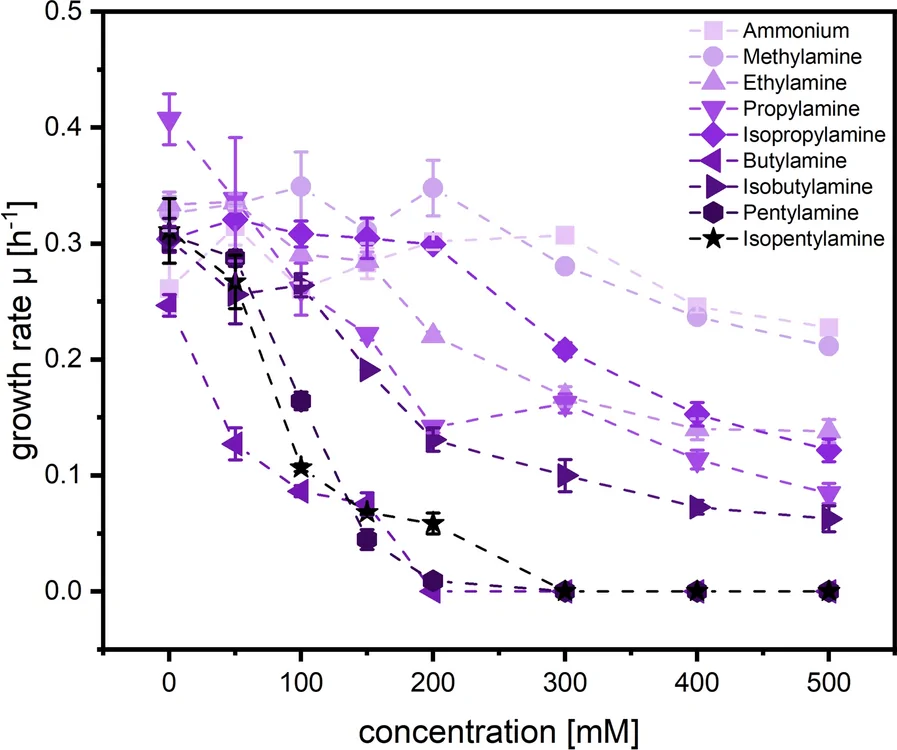
Dependence of growth rates of *C. glutamicum* WT on different SCPAs with an alkyl chain length ≤ 5 carbons tested at concentrations between 0 and 500 mM

For the effective production of SCPAs a high tolerance toward these reactive compounds is beneficial. Therefore, growth of *C. glutamicum* with a variety of linear and branched SCPAs was analyzed. Cultivations were performed in Duetz plates with CGXII 4% glucose production media. In general, the toxicity increased with the alkyl chain length of the amine (Fig. 1); the only exception of which was isopropylamine with *K*_i_ = 0.4 M, twice as high as for its linear isomer propylamine with *K*_i_ = 0.2 M. No toxic effect was found for ammonium in the tested concentration range, while the addition of 100 mM of the C5 isomers pentylamine and isopentylamine reduced the growth rate to half-maximal. The *K*_i_ value of 0.23 M for isobutylamine indicated high tolerance of *C. glutamicum* toward this compound and, therefore, suitability as a production host for this compound.

### Decarboxylation of l-valine to yield isobutylamine in vitro

**Fig. 2:**
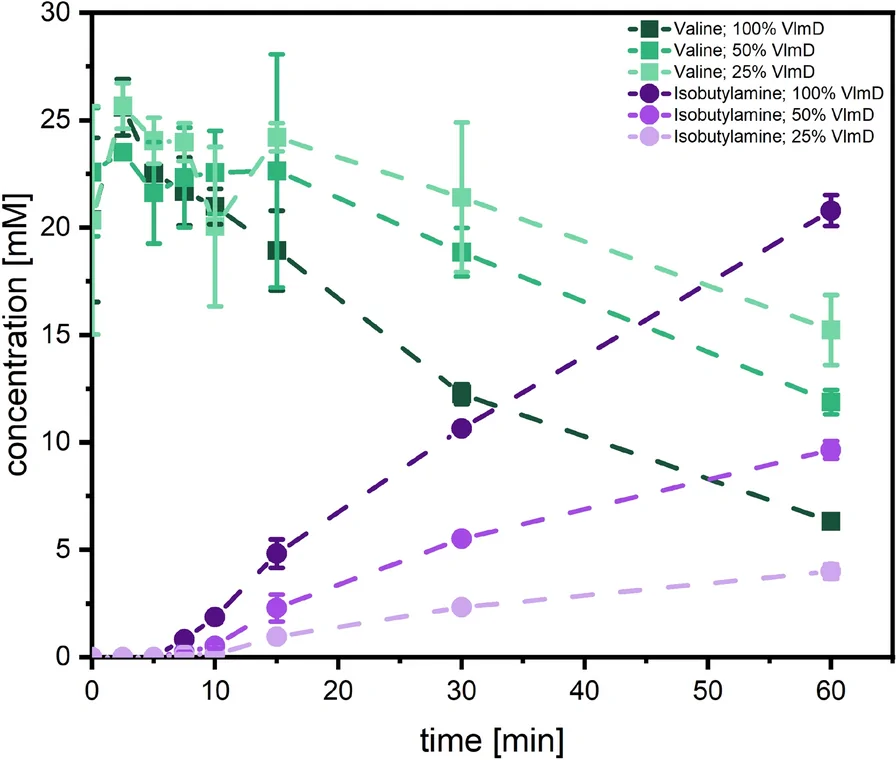
Isobutylamine formation by l-valine decarboxylation in crude extracts from *E. coli* BL21(DE3) (pRG_Duet3–*vlmD*_*Sv*_) with 20 mM l-valine as substrate. l-valine (green) and isobutylamine (violet) concentrations from 0 to 60 min are shown. The assay was performed with different dilutions of protein concentrations (100%; 50% and 25%). Darker colors indicate higher protein concentrations. Squares and dots indicate mean values and error bars indicate arithmetic deviation, n = 2. Crude extracts from the empty vector carrying control did not show isobutylamine formation

To construct an isobutylamine-producing strain, the l-valine decarboxylase from *Streptomyces viridifaciens* (VlmD_Sv_) was used, since decarboxylation reactions benefit from CO_2_ outgassing. The suitability of this enzyme was evaluated in a crude extract assay. Cell free extract of *E. coli* BL21(DE3) (pRG_Duet3–*vlmD*_*Sv*_) and the respective empty vector control were incubated for 60 min under conditions relevant for *C. glutamicum*: pH 7 and 30 °C with 20 mM l-valine as substrate. Crude extracts from the empty vector carrying control did not show isobutylamine formation. Isobutylamine was first detected by HPLC analysis after 7.5 min of incubation for all protein concentrations (Fig. 2). The concentrations of isobutylamine increased and the l-valine decreased for subsequent timepoints. After incubation for 60 min, final isobutylamine concentrations of 20.8 ± 0.7 mM (100%), 9.7 ± 0.4 mM (50%) and 4.0 ± 0.3 mM (25%) were measured. The l-valine concentrations were 6.3 ± 0.3 mM (100%), 11.9 ± 0.6 mM (50%) and 15.2 ± 1.6 mM (25%), respectively. The activity of the crude extract was calculated from the linear regression of the isobutylamine concentrations from 7.5 min to 60 min as 0.4 ± 0.1 U mg^−1^. Thus, VlmD_Sv_ appeared to be suitable for isobutylamine production in vivo.

### Establishing isobutylamine production by expression of *vlmD*_*Sv*_ in three pyruvate-producing strains

**Fig. 3:**
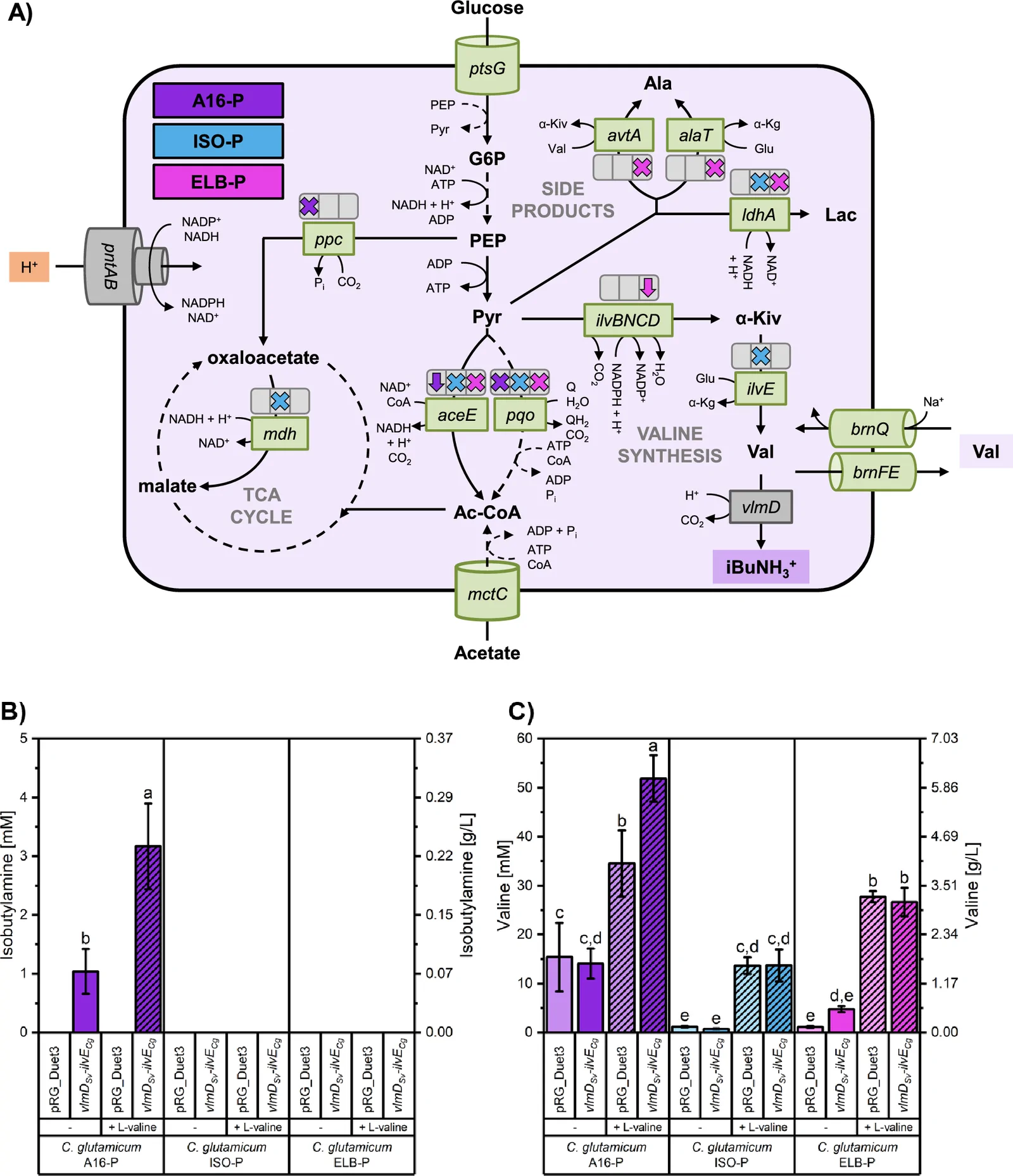
Schematic metabolic overview of isobutylamine-producing strains (**A**). Deletions are shown as crosses, downregulations as downward-pointing arrows in gray boxes above or below the respective gene for each strain: *C. glutamicum* ISO-P (blue), ELB-P (pink) and A16-P (violet). Native genes are shown in green, non-native genes as gray. Metabolites: acetyl-coenzyme A (Ac-CoA), α-ketoglutarate (α-Kg), α-ketoisovalerate (α-Kiv), l-alanine (Ala), glucose-6-phosphate (G6P), l-glutamate (Glu), isobutylamine (protonated, iBuNH_3_), lactate (Lac), phosphoenolpyruvate (PEP), pyruvate (Pyr), quinone (Q; QH2), l-valine (Val). Genes: pyruvate dehydrogenase E1 subunit (*aceE*), glutamate-dependent pyruvate:L-alanine transaminase (*alaT*), l-valine dependent pyruvate:l-alanine transaminase (*avtA*), BCAA exporter (*brnFE*), BCAA importer (*brnQ*), malate dehydrogenase (*mdh*), monocarboxylic acid transporter (*mctC*), phosphoenolpyruvate carboxylase (*ppc*), proton-translocating NAD(P)^+^ transhydrogenase (*pntAB*) pyruvate:quinone oxidoreductase (*pqo*), phosphotransferase system glucose-specific permease (*ptsG*), lactate dehydrogenase (*ldhA*), acetolactate synthase (*ilvBN*), ketol acid reductoisomerase (*ilvC*), dihydroxy acid dehydratase (*ilvD*), BCAA transaminase (*ilvE*), l-valine decarboxylase (*vlmD*). Isobutylamine (**B**) and l-valine titers (**C**) obtained after 48 h of cultivation are given in mM (left *y*-axis) and g/L (right *y*-axis). Black stripes indicate addition of l-valine. Error bars indicate standard deviation, n = 3

Three pyruvate-producing strains were chosen as basis for the production of isobutylamine (Fig. 3A). The key enzyme in these strains is the pyruvate dehydrogenase for the conversion of pyruvate to acetyl-CoA competing with the BCAA synthesis. Strains ELB-P and ISO-P carry a deletion of the *aceE* gene and were, therefore, acetate auxotrophic, while *aceE* was downregulated by a chromosomal promoter exchange in strain A16-P. In these strains, glycolysis was decoupled from the TCA cycle, allowing for growth-decoupled production of pyruvate from glucose and growth from acetate [6]. The pyruvate:quinone oxidoreductase gene *pqo* was deleted in all strains to eliminate a possible bypass of the *aceE* deletion. A deletion of the *ppc* gene reduced anaplerotic flux into the TCA cycle in strain A16-P. Furthermore, to reduce byproduct formation, ELB-P and ISO-P have a deleted lactate dehydrogenase gene *ldhA;* in addition, the pyruvate:l-alanine transaminase genes *alaT* and *avtA* are deleted in ELB-P, leading to an l-alanine auxotrophy. ISO-P carries a deletion of the malate dehydrogenase gene to reduce succinate byproduct formation by reductive TCA cycle activity. The high production capabilities of compounds structurally related to isobutylamine have been demonstrated before in the selected strains. ELB-P has previously been utilized as a dedicated pyruvate producer strain. An ISO-P derivative has been engineered for isobutanol production, and an A16-P-derived strain has been used to produce l-valine [5, 8, 53]. The production of branched-chain amino acids, which are the direct precursors for the production of SCPAs by the l-valine decarboxylase relies on the *ilvBNC* operon*, ilvD* and *ilvE* starting from pyruvate as substrate. Conversion by IlvC requires NADPH; therefore, the proton-translocating NAD(P)^+^ transhydrogenase PntAB from *E. coli* was overexpressed to increase NADPH supply, as previously shown to be beneficial for the production of isobutanol with ISO-P [5].

The isobutylamine production plasmid pRG_Duet3–*vlmD*_*Sv*_–*ilvE*_*Cg*_ was constructed and used to transform the three pyruvate producer strains. The plasmid-based *ilvE*_*Cg*_ overexpression was added to compensate for the corresponding deletion in ISO-P. The strains were then cultivated on CGXII media with 4% glucose and 1% K-acetate, isobutylamine and l-valine concentrations were quantified and growth parameters determined (Fig. 3B–D). For the l-valine bradythrophic strains ISO-P and ELB-P, 2 mM l-valine was added. To investigate whether increased precursor supply leads to higher isobutylamine production, all strains were also cultivated with addition of 20 mM l-valine. A16-P (pRG_Duet3–*vlmD*_*Sv*_–*ilvE*_*Cg*_) produced 1.1 ± 0.4 mM isobutylamine without addition of l-valine. l-valine addition increased the titer by ~threefold to 3.2 ± 0.7 mM. Neither the corresponding empty vector strain, nor ISO-P or ELB-P-derived strains showed any detectable isobutylamine production. Significant amounts of l-valine were secreted by the same strain that produced isobutylamine. Without additional l-valine, A16-P (pRG_Duet3–*vlmD*_*Sv*_–*ilvE*_*Cg*_) produced 14.1 ± 3.1 mM l-valine, the same as the empty vector strain with 15.4 ± 7 mM. However, when 20 mM l-valine were added, disproportionally more l-valine with 51.9 ± 4.8 mM were detected, significantly more than in the empty vector strain with 34.5 ± 6.8 mM, for which the concentration increased proportionally. For ISO-P and ELB-P, the amount that was expected from the addition of either 2 mM l-valine or 20 mM l-valine was found in the supernatant, indicating no significant l-valine production or consumption in these strains. Since the l-valine addition increased the isobutylamine and l-valine production in A16-P and no significant concentration of either l-valine or isobutylamine was produced by ISO-P and ELB-P, we aimed to increase the precursor supply.

### Metabolic engineering for increased precursor supply

To increase the intracellular l-valine availability for decarboxylation by the heterologously expressed l-valine decarboxylase, two strategies were applied. First, the BCAA production plasmid pJC4*ilvBNCD*_*Cg*_–*pntAB*_*Ec*_ for the overexpression of the l-valine synthesis genes and *pntAB*_*Ec*_ for increased NADPH supply was introduced into the strains that carried the l-valine decarboxylation plasmid pRG_Duet3–*vlmD*_*Sv*_–*ilvE*_*Cg*_. Second, to reduce the l-valine secretion into the medium, the BCAA exporter genes *brnFE*_*Cg*_ were deleted. The resulting strains were cultivated under the same conditions as before. Their supernatant was analyzed via HPLC measurement.

**Fig. 4:**
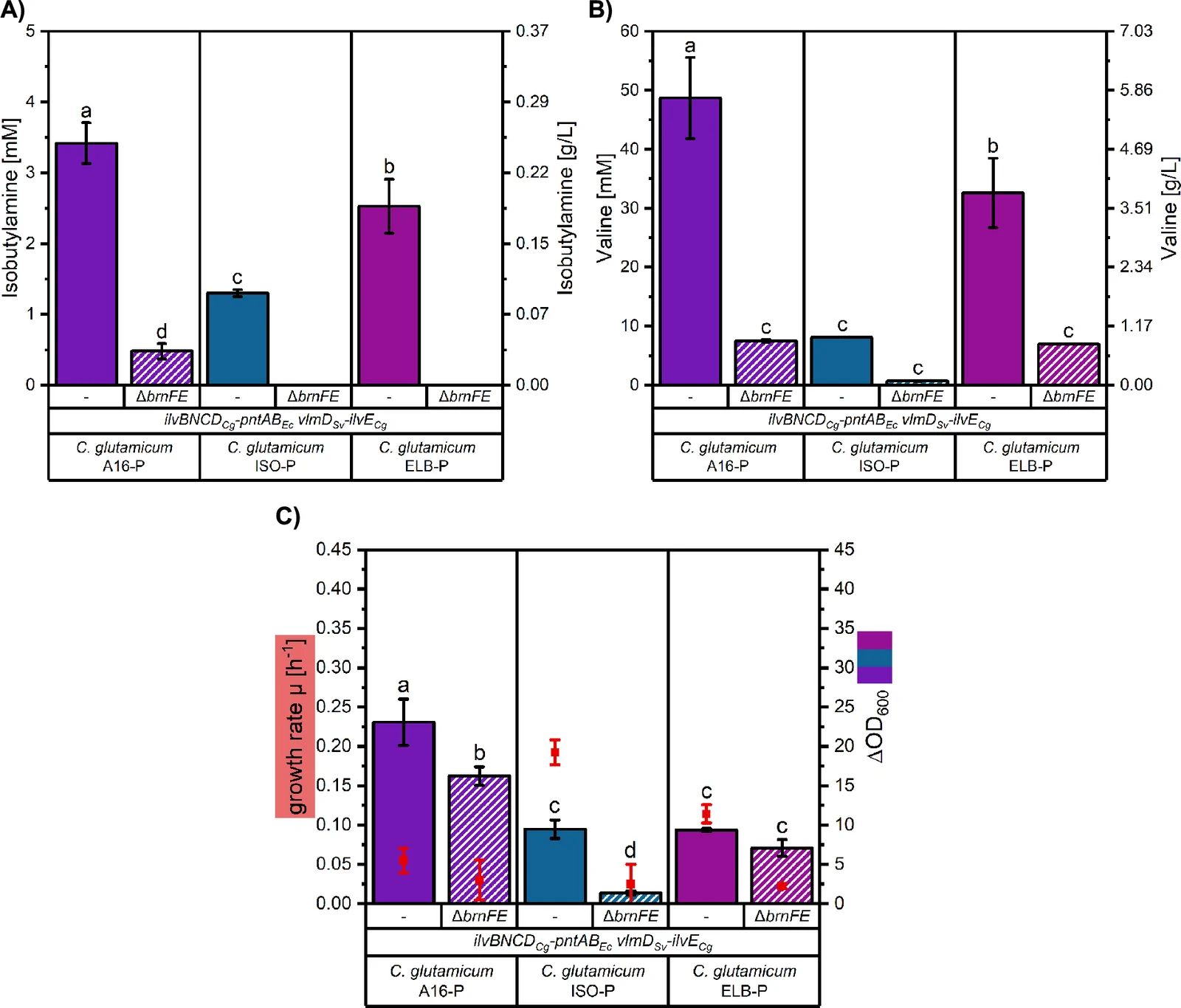
Isobutylamine production by various strains engineered for increased precursor (l-valine) supply for decarboxylation to isobutylamine. Isobutylamine (**A**) and l-valine (**B**) titers after 48 h of cultivation in mM (left *y*-axis) and g/L (right *y*-axis). The mean growth rates (red squares) and mean ΔOD_600_ after 48 h (columns in respective strain color) were determined (**C**). Black stripes indicate addition of l-valine, white stripes indicate Δ*brnFE*. Error bars indicate standard deviation, n = 3

The addition of the BCAA production plasmid increased the isobutylamine and l-valine titers significantly after 48 h of cultivation (Fig. 4A, B). For the A16-P-derived strain, the isobutylamine and l-valine titers increased approximately threefold to 3.4 ± 0.3 mM and 48.7 ± 6.9 mM, respectively (Fig. 4A). The ISO-P-derived strain accumulated significantly more metabolites with 1.3 ± 0.05 mM isobutylamine and 8.1 ± 0.03 mM l-valine. For the ELB-P-derived strain, 2.5 ± 0.4 mM isobutylamine and 32.6 ± 5.9 mM l-valine were detected in the supernatant, marking a significant increase. Therefore, in the Δ*aceE* strains, the overexpression of *ilvBNCD*_*Cg*_ was necessary for the production of isobutylamine to compensate for the deletions that were introduced in the parental strains to reduce l-valine byproduct. The titers increased slightly, non-significantly after 72 h of cultivation (data not shown). The strains derived from the two Δ*aceE* strains reached a ΔOD_600_ of approximately 9.4 (Fig. 4C), while the A16-P-based strain reached a ΔOD_600_ of 23.1 ± 2.9, due to it being able to still feed into the TCA cycle from glycolysis.

Expectedly, the deletion of the l-valine exporter genes *brnFE* significantly reduced the extracellular l-valine accumulation (Fig. 4B). For all strains the concentration was reduced approximately six- to sevenfold. The A16-P-derived strain produced 7.5 ± 0.25 mM, the ISO-P strain accumulated 0.7 ± 0.06 mM and the ELB-P strain 7 ± 0.02 mM l-valine. However, the isobutylamine titers were also significantly reduced. While A16-P Δ*brnFE* (pJC4*ilvBNCD*_*Cg*_–*pntAB*_*Ec*_) (pRG_Duet3–*vlmD*_*Sv*_–*ilvE*_*Cg*_) was able to produce 0.5 ± 0.1 mM isobutylamine, a sevenfold decrease, the corresponding ISO-P and ELB-P strains did not produce any isobutylamine. The growth rates and ΔOD_600_ decreased significantly for all strains as well, indicating increased stress, possibly due to increased intracellular l-valine accumulation upon deletion of *brnFE*_*Cg*_.

### Influence of heterologous PntAB expression on isobutylamine production and on the membrane potential

In all strains analyzed until here, *pntAB*_*Ec*_ was overexpressed as it was expected to increase NADPH supply. This has previously been shown to be beneficial for the anaerobic production of isobutanol with strain ISO-P [5]. However, isobutylamine titers observed here were drastically lower than isobutanol titers [5] although obtained with the same strain background, i.e., ISO-P. Thus, we concluded that expression of *pntAB*_*Ec*_ may be not beneficial. Therefore, the *pntAB*_*Ec*_ containing plasmids were exchanged with either the pJC4 empty vector, pJC4*ilvBNCD*_*Cg*_ or pJC4*ilvBNCD*_*Cg*_–*leuA*_*Cg*_^G462D^ in the A16-P-derived isobutylamine production strains (Table 1). In addition, since the deletion of the l-valine exporter BrnFE_Cg_ did not have a positive effect on the production in the former previous cultivations, we overexpressed the l-valine importer gene *brnQ*_*Cg*_, to reimport secreted l-valine from the supernatant. For this purpose, the plasmid pECXT99A–*vlmD*_*Sv*_–*brnQ*_*Cg*_ and the control plasmid pECXT99A–*vlmD*_*Sv*_ were constructed.

**Fig. 5:**
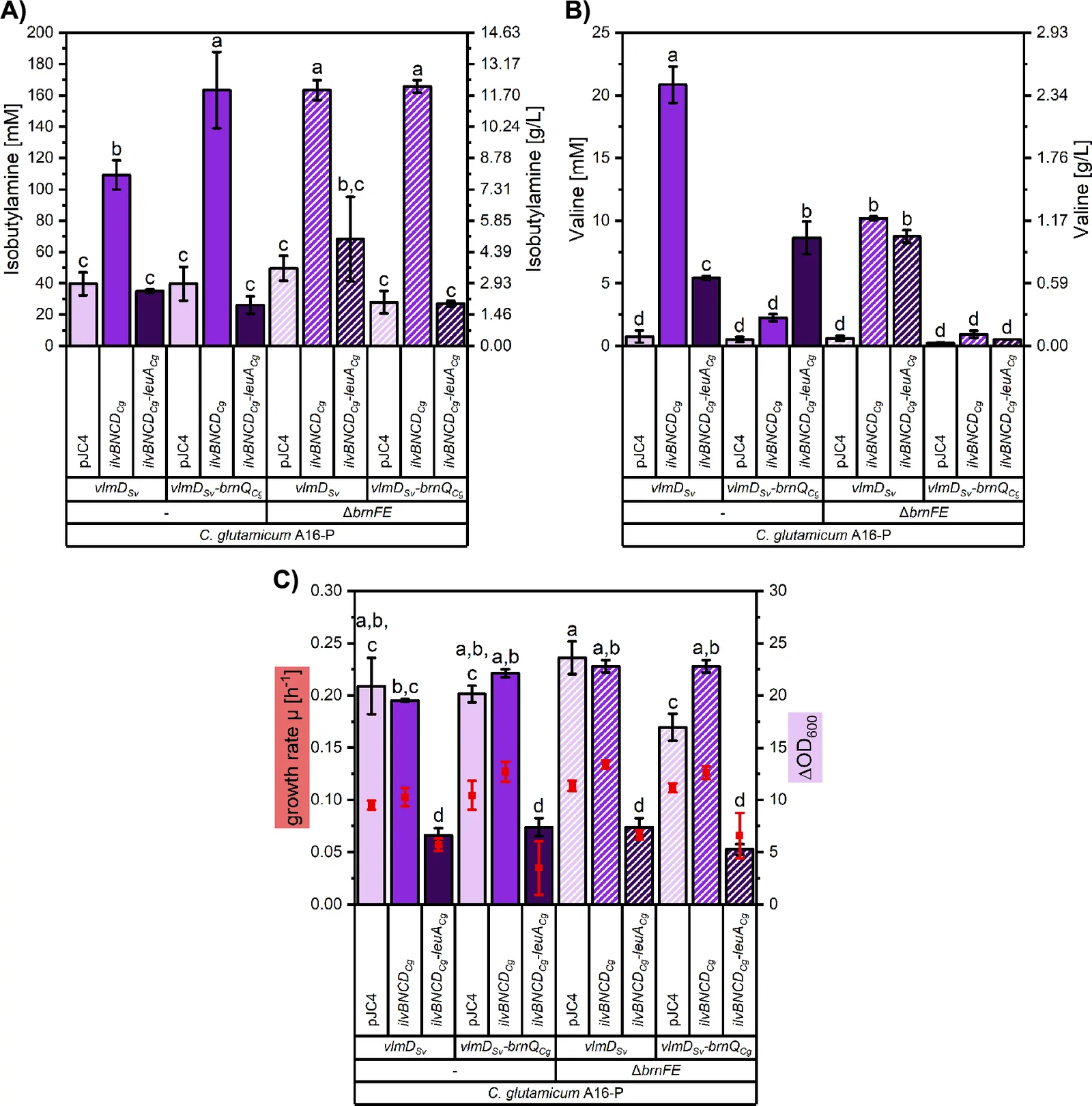
Influence of overexpression of biosynthesis genes as well as BCCA export system BrnFE_Cg_ and the BCCA importer BrnQ_Cg_ on isobutylamine production by strain A16-P. *C. glutamicum* A16-P-derived strains carried a deletion of the gene for the BCAA export system BrnFE_Cg_, overexpressed the gene for the BCAA importer BrnQ_Cg_, the *ilvBNCD*_*Cg*_ biosynthesis operon and/or the *leuA*_*Cg*_^G462D^ gene and various combinations of these genetic modifications. Isobutylamine (**A**); l-valine (**B**) titers after 72 h of cultivation in mM (left *y*-axis) and g/L (right *y*-axis). The mean growth rates (red squares) and mean ΔOD_600_ after 72 h (columns in respective strain color) were determined (**C**). Black stripes indicate addition of l-valine, white stripes indicate Δ*brnFE*. Error bars indicate standard deviation, n = 3

The growth was similar for all strains, except the *leuA*_*Cg*_^G462D^ strains (Fig. 5C), that grew significantly slower and to lower final ODs. After 48 h and 72 h of cultivation, the pJC4*ilvBNCD*_*Cg*_ strains showed significantly higher isobutylamine production than the other strains. Surprisingly, the production ranged between ~ 120 mM and ~ 140 mM after 48 h of cultivation (data not shown). The deletion of *brnFE*_*Cg*_ and the overexpression of *brnQ*_*Cg*_ led to significantly increased titers; however, the combination of both led to no further additive increase. The highest production titers were detected after 72 h (Fig. 5A) for A16-P (pJC4*ilvBNCD*_*Cg*_) (pECXT99A–*vlmD*_*Sv*_–*brnQ*_*Cg*_) with 163.5 ± 24.3 mM; A16-P Δ*brnFE* (pJC4*ilvBNCD*_*Cg*_) (pECXT99A–*vlmD*_*Sv*_) with 163.4 ± 6.3 mM and A16-P Δ*brnFE* (pJC4*ilvBNCD*_*Cg*_) (pECXT99A–*vlmD*_*Sv*_–*brnQ*_*Cg*_) with the highest titer of 165.7 ± 4 mM. In pJC4*ilvBNCD*_*Cg*_ carrying strains the extracellular l-valine concentrations decreased significantly upon deletion of *brnFE*_*Cg*_ or overexpression of *brnQ*_*Cg*_ (Fig. 5B). The l-valine titer decreased from 20.9 ± 1.4 mM 9.5-fold to 2.2 mM upon overexpression of *brnQ*_*Cg*_ and halved to 10.2 ± 0.1 mM when *brnFE*_*Cg*_ was deleted. For the strain that combined the *brnFE*_*Cg*_ deletion and *brnQ*_*Cg*_ overexpression, 0.9 ± 0.3 mM l-valine were detected. These findings indicate that the isobutylamine titer increased due to improved intracellular availability of l-valine. The isobutylamine titers of the *leuA* strains were in a similar range as the empty vector strains, indicating that the benefit of the *ilvBNCD*_*Cg*_ overexpression for the isobutylamine production is negated by the *leuA*_*Cg*_^G462D^ expression. This may be due to the fact that glucose was not utilized completely by *leuA* strains, but by all other strains. We speculated that isobutylamine affects the transmembrane proton motif force. The membrane potential of *C*. gl*utamicum* WT samples in the exponential growth phase was analyzed by addition of DiOC_2_(3) and subsequent sorting by FACS (Fig. 7A). Isobutylamine was added at the beginning of the cultivation or directly before the measurement. Samples treated with 50 µM CCCP served as positive control for a decoupled membrane potential. When cells in the exponential growth phase after approximately 6 h of cultivation were treated with 100 mM isobutylamine directly before staining, the FACS event rate was reduced immediately in comparison with the untreated control cells as well as a decreased red/green fluorescence ratio, indicating that the cells were killed by a decoupled transmembrane potential upon addition of isobutylamine. Cultures grown with addition of 50 mM or 100 mM isobutylamine from the beginning of the cultivation exhibited an increased membrane potential relative to the 0 mM negative control. However, when 200 mM were added initially, a lower overall OD_600_ and a reduction of transmembrane potential were detected.

**Fig. 6:**
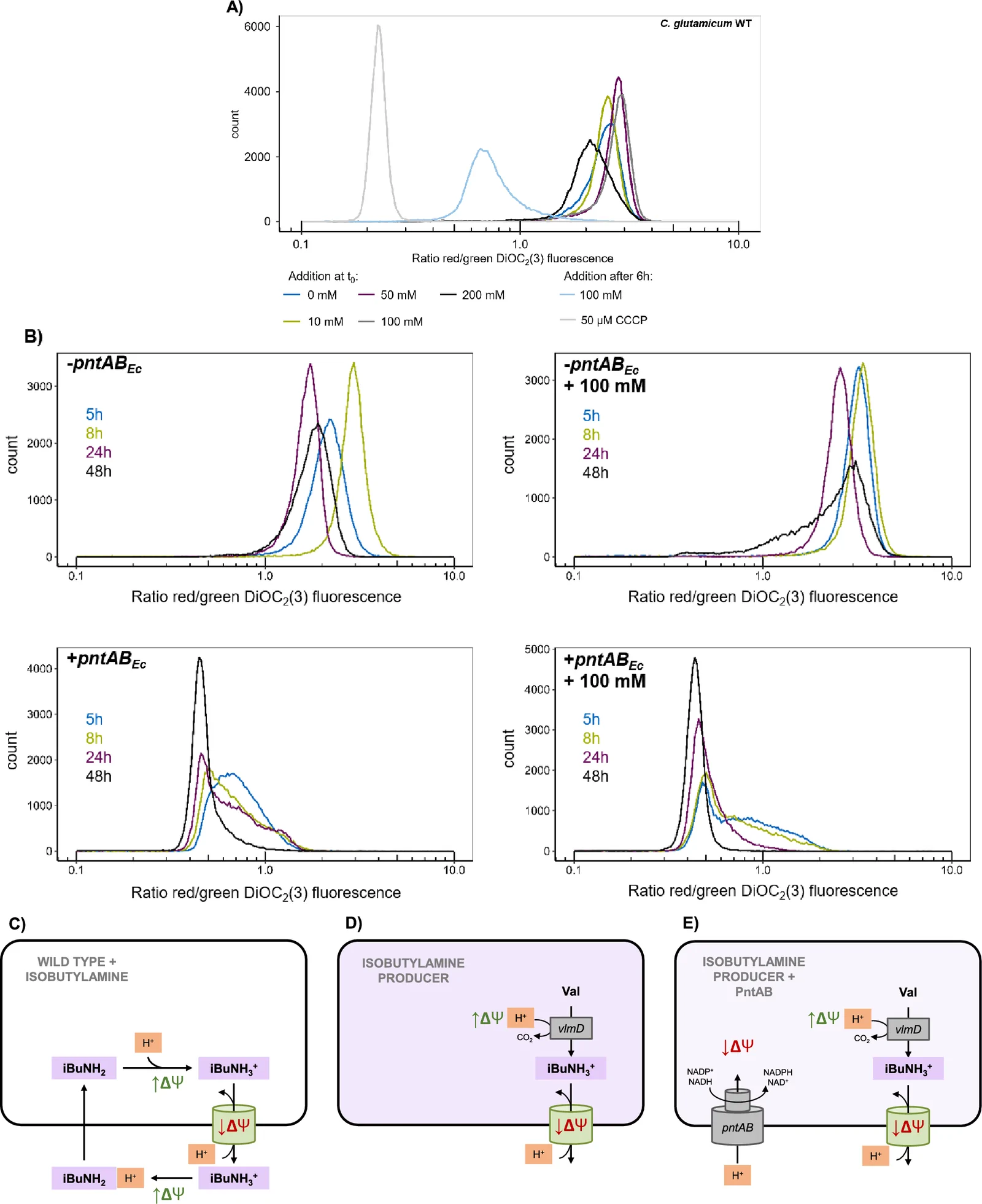
Influence of externally added and intracellularly produced isobutylamine in the presence or absence of *pntAB*_*Ec*_ on the membrane potential. Relative comparison of the membrane potential of *C. glutamicum* WT cultivated with different concentrations of isobutylamine for 6 h indicated as the ratio of red/green DiOC_2_(3) fluorescence (**A**). Relative comparison of the membrane potential of the best producing strain A16-P Δ*brnFE* (pJC4*ilvBNCD*_*Cg*_) (pECXT99A–*vlmD*_*Sv*_–*brnQ*_*Cg*_) with and without overexpression of *pntAB*_*Ec*_ and with or without addition of 100 mM isobutylamine throughout their cultivation indicated as the ratio of red/green DiOC_2_(3) fluorescence (**B**). Schematic interpretation of potential mechanisms leading to changes of the membrane potential (∆ψ) in the wild-type strain upon addition of extracellular isobutylamine (**C**) as well as during isobutylamine production of the producer strain without *pntAB*_*Ec*_ expression (**D**) or with *pntAB*_*Ec*_ expression (**E**)

The untreated cells showed a decreased fluorescence ratio and, therefore, membrane potential in the stationary phase (data not shown). Cultures with 50 mM and 100 mM isobutylamine showed an increased membrane potential, possibly hyperpolarization, while cultivation with 200 mM led to a reduction relative to the control. When 20 mM isobutylamine was added to untreated cells (diluted to OD 0.05), the FACS event rate was reduced immediately and the fluorescence ratio decreased, indicating that cells were killed by a decoupled transmembrane potential. *C. glutamicum* exhibited a higher tolerance toward isobutylamine when it was added from the beginning of the cultivation than when it was added later. Since the transhydrogenase reaction catalyzed by PntAB_Ec_ is coupled to the proton motif force, the H^+^ transmembrane gradient was analyzed as described before. The best producer strain *C. glutamicum* A16-P (pJC4*ilvBNCD*_*Cg*_) (pECXT99A–*vlmD*_*Sv*_–*brnQ*_*Cg*_) and the respective +*pntAB*_*Ec*_ strain were analyzed for differences in their transmembrane gradient during cultivation conditions and with or without addition of isobutylamine (Fig. 6B). The transmembrane potential of the -*pntAB*_*Ec*_ strain increased during the first 8 h of cultivation and decreased from there after 24 h, with and without addition of isobutylamine, where the addition had a slight polarizing effect. The +*pntAB*_*Ec*_ strain showed a stronger depolarization during cultivation than the -*pntAB*_*Ec*_ strain. Supplementation of 100 mM isobutylamine at t_0_ led to increased depolarization. The growth of the +*pntAB*_*Ec*_ strain was significantly slower and to a lower final OD than the -*pntAB*_*Ec*_ strain (data not shown).

### Production of 3-methylbutan-1-amine by exploiting VlmD substrate promiscuity

**Fig. 7:**
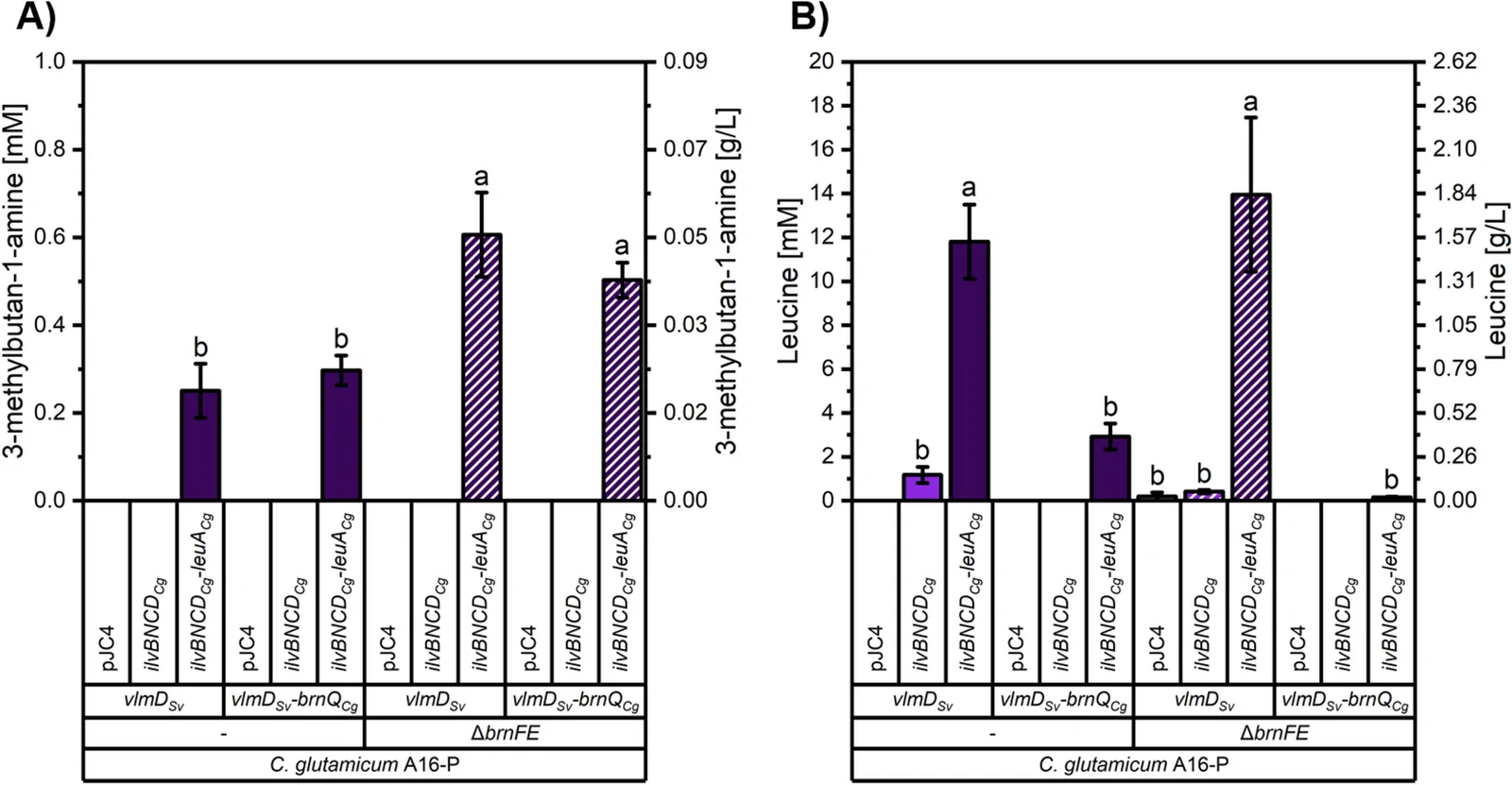
3-Methylbutan-1-amine production by various strains. 3-Methylbutan-1-amine (**A**) and l-leucine (**B**) titers after 72 h of cultivation are given in mM (left *y*-axis) and g/L (right *y*-axis). Black stripes indicate addition of l-valine, white stripes indicate Δ*brnFE*. Error bars indicate standard deviation, n = 3

VlmD_Sv_ is a promiscuous enzyme with broad substrate spectrum, with the highest affinity toward l-valine [24]. Therefore, *vlmD*_*Sv*_ was expressed in strains in which the l-leucine production plasmid pJC4*ilvBNCD*_*Cg*_–*leuA*_*Cg*_^G462D^ was used instead of the l-valine production plasmid. The feedback resistant variant of the *leuA*_*Cg*_^G462D^ gene was expected to redirect the flux from l-valine toward l-leucine biosynthesis. After 72 h of cultivation all pJC4*ilvBNCD*_*Cg*_–*leuA*_*Cg*_^G462D^ produced 3-methylbutan-1-amine, while the pJC4 empty vector strains and the pJC4*ilvBNCD*_*Cg*_ strains did not show any 3-methylbutan-1-amine production (Fig. 7A). The overexpression of *brnQ*_*Cg*_ did not increase the production of 3-methylbutan-1-amine significantly as A16-P (pJC4*ilvBNCD*_*Cg*_–*leuA*_*Cg*_^G462D^) (pECXT99A–*vlmD*_*Sv*_) and the corresponding *brnQ*_*Cg*_ overexpression strain produced 0.25 ± 0.06 mM and 0.3 ± 0.03 mM, respectively. Deleting the exporter genes *brnFE*_*Cg*_ increased the titer significantly to 0.61 mM ± 0.1 mM, while the additional overexpression of *brnQ*_*Cg*_ did not change the titer significantly. The l-leucine titers were significantly increased by the overexpression of *ilvBNCD*_*Cg*_–*leuA*_*Cg*_^G462D^_._ All strains with the exception of *leuA*_*Cg*_^G462D^ overexpression strains produced less than 1 mM l-leucine (Fig. 7B). Strain A16-P (pJC4*ilvBNCD*_*Cg*_–*leuA*_*Cg*_^G462D^) (pECXT99A–*vlmD*_*Sv*_) secreted 11.8 ± 1.7 mM l-leucine, while no l-leucine was detected for the respective pJC4 empty vector strain. The additional overexpression of *brnQ*_*Cg*_ reduced the titer to 2.9 ± 0.6 mM, possibly increasing intracellular l-leucine levels, while the deletion of *brnFE*_*Cg*_ did not change the extracellular concentration significantly.

### Production of isobutylamine on second-generation feedstocks

**Fig. 8:**
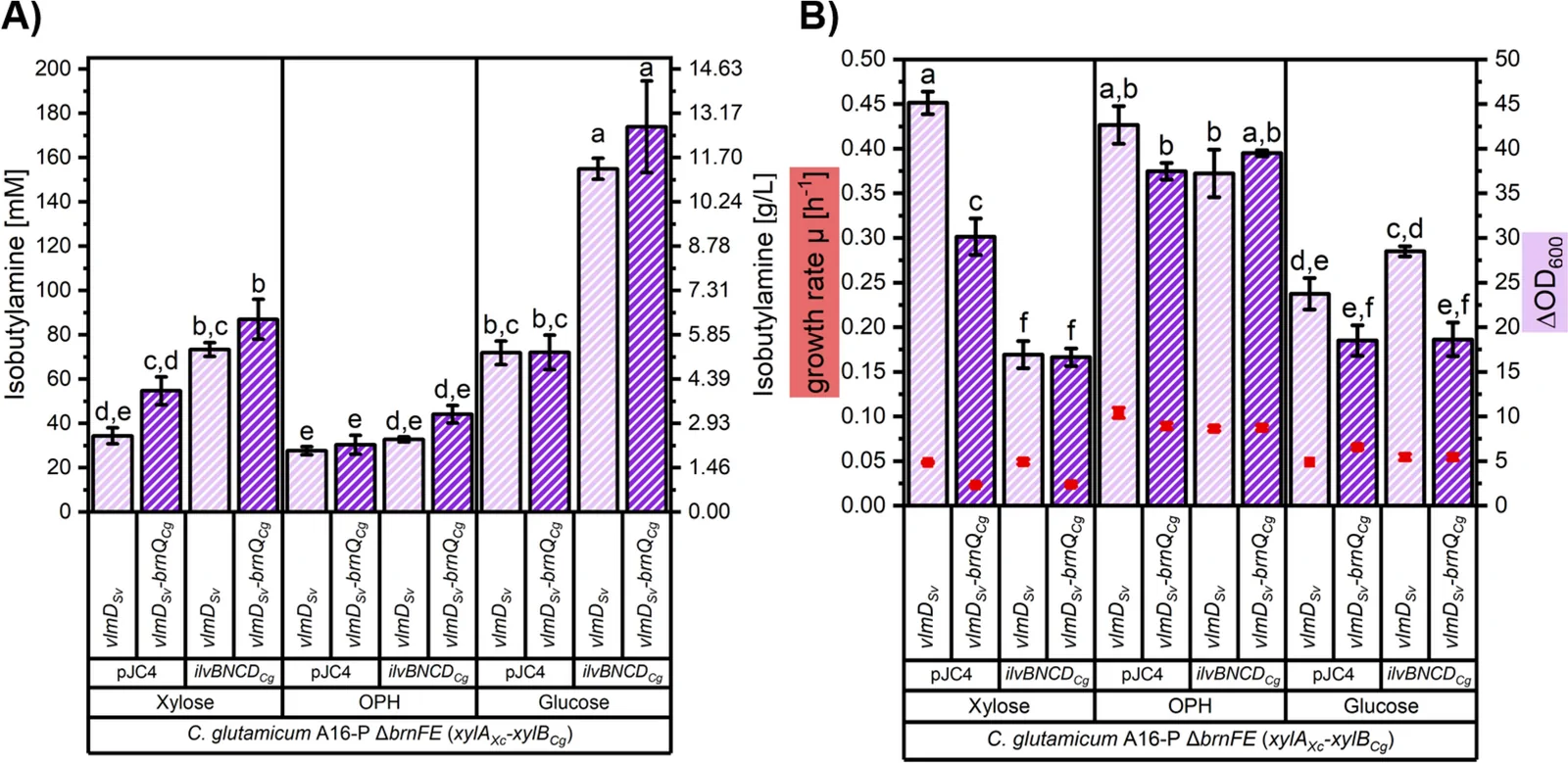
Isobutylamine production on second-generation feedstocks xylose and orange peel hydrolysate. Isobutylamine product titers after 120 h of cultivation are given in mM (left *y*-axis) and g/L (right *y*-axis) (**A**). The mean growth rates (red squares) and mean ΔOD_600_ after 72 h are given (**B**). Light columns indicate expression of VlmD_Sv_ alone, while darker columns indicate the additional expression of BrnQ_Cg_, white stripes indicate Δ*brnFE*. Error bars indicate standard deviations, n = 3

To demonstrate the feasibility of isobutylamine production on second-generation feedstocks, the plasmid pEKEx3–*xylA*_*Xc*_*B*_*Cg*_ was added to the best producers and their respective pJC4 empty vector controls. This allowed utilization of the lignocellulosic pentose xylose or xylose-containing orange peel hydrolysate. The xylose utilizing producer strains were cultivated in CGXII with 40 g/L glucose as control. In addition, either 40 g/L xylose was used or orange peel hydrolysate, that was prepared as described before [21, 46] and which was adjusted to a concentration of 24.2 ± 2.9 g/L glucose and 15.9 ± 1.6 g/L xylose. The strains were fully grown after 120 h of cultivation. The highest growth rates and ΔOD_600_ were reached on orange peel hydrolysate indicating that it is a good substrate to support growth (Fig. 8B). The control cultivation with glucose led to the highest titers with 173.9 ± 20.7 mM isobutylamine (Fig. 8A), similar as observed before (Table 1, Fig. 7A). With xylose as sole carbon source, a maximum of 87 ± 9 mM isobutylamine was produced by strain A16-P (pJC4*ilvBNCD*_*Cg*_) (pECXT99A–*vlmD*_*Sv*_–*brnQ*_*Cg*_) (pEKEx3–*xylA*_*Xc*_*B*_*Cg*_). Up to 44.2 ± 3.9 mM isobutylamine was produced by the same strain during cultivation with orange peel hydrolysate. Thus, isobutylamine production on xylose and orange peel hydrolysate was successful, but since the titer on glucose was higher, future work must address process optimization for second-generation hydrolysate-based isobutylamine production processes.

### Lab-scale fed-batch bioreactor cultivation for isobutylamine production

**Fig. 9:**
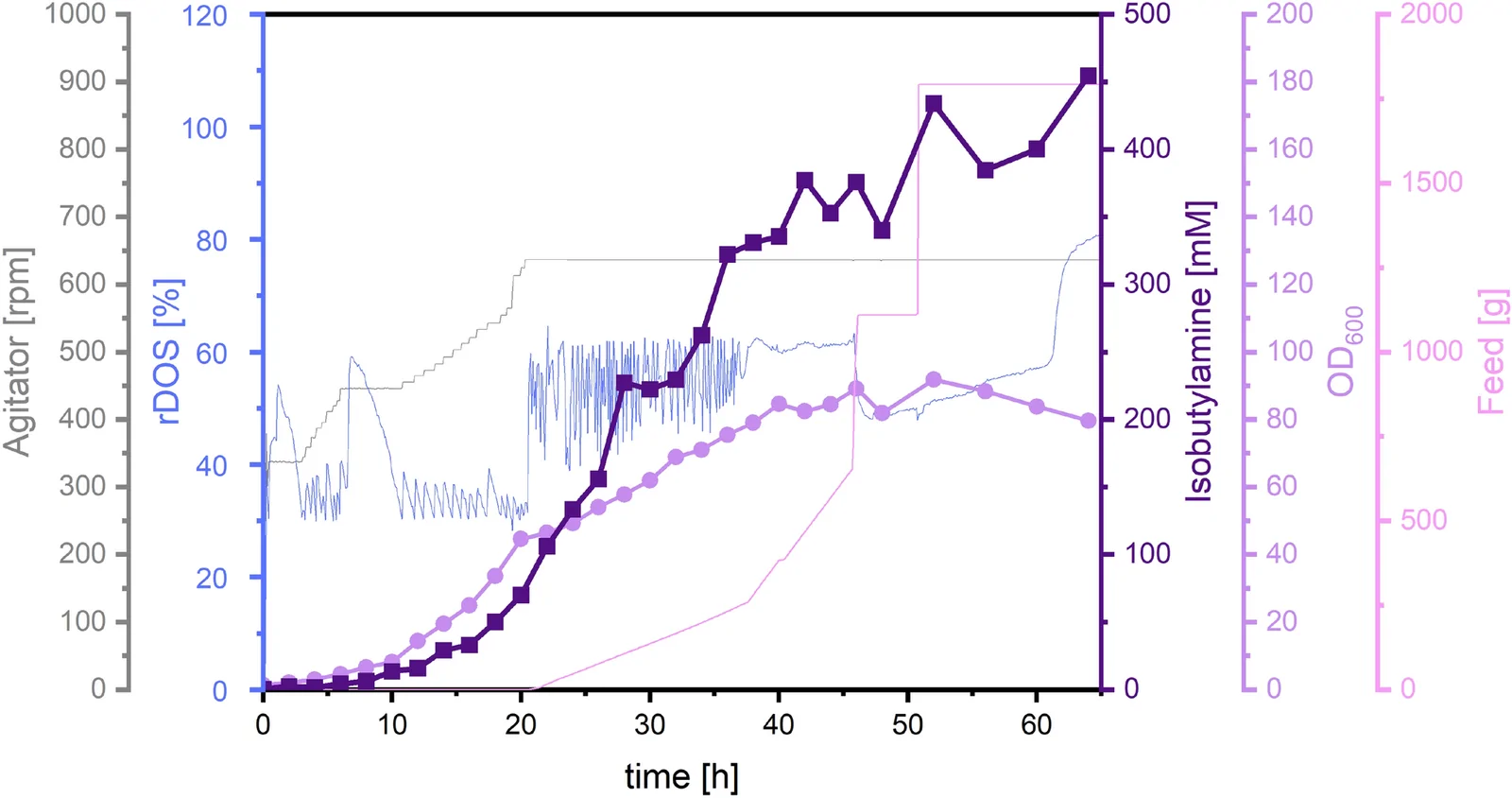
Isobutylamine production in 3 L fed-batch fermentation. Strain A16-P Δ*brnFE* (pJC4*ilvBNCD*_*Cg*_) (pECXT99a–*vlmD*_*Sv*_–*brnQ*_*Cg*_) was cultivated in CGXII medium pH 7.0 with 4% glucose at 30 °C. 1 L 40% glucose with additional trace elements, biotin, PCA, 1 mM IPTG, CaCl_2_, MgSO_4_ were used as feed. The isobutylamine titers were calculated by using the initial volume of 2 L

To test if the production is stable at higher volumes, isobutylamine production was tested in a 3 L fed-batch bioreactor fermentation. Strain A16-P Δ*brnFE* (pJC4*ilvBNCD*_*Cg*_) (pECXT99A–*vlmD*_*Sv*_–*brnQ*_*Cg*_) as best and most stable isobutylamine producer was cultivated for 64 h with glucose as sole carbon source and reached a maximum titer of 454 mM (33.2 g/L) isobutylamine (Fig. 9) with a *Y*_p/s_ 0.47 mol/mol and a volumetric productivity *r*_p_ of 0.52 g/L/h based on the initial volume. About 357 mM glucose remained in the supernatant after fermentation. Major byproducts were l-alanine (~ 43 mM) and BCAAs (~ 15 mM). A nonsignificant amount of isobutylamine was detected in the off-gas. Since the isobutylamine titer almost tripled from 166 mM (12.1 g/L) in shake flask cultivation to 454 mM (33.2 g/L) isobutylamine in fed-batch bioreactor fermentation, the process benefited from the upscaling.

## Discussion

Pathway extension from pyruvate to isobutylamine including l-valine decarboxylase VlmD_Sv_ enabled isobutylamine production, which was further increased upon transport engineering (abolishing l-valine export and increasing its re-uptake). Isobutylamine production was achieved using second-generation feedstocks and was scaled to 3 L fed-batch bioreactor fermentation with a titer of 33.2 g/L, a yield of 0.47 mol% and a volumetric productivity of 0.52 g/L/h.

l-valine supply for VlmD_Sv_ was improved by reducing its export and by increasing its reuptake. As has been reported before, *brnFE*_*Cg*_ deletion in BCAA overproducing strains impaired growth due to accumulation of BCAAs to high intracellular concentrations [22]. BrnFE_*Cg*_ exports l-methionine in addition to BCAAs and expression of the *brnFE*_*Cg*_ operon is controlled by Lrp_Cg_ [27]. Overexpression of *lrp*_*Cg*_ improved l-isoleucine production [55]. Production of 4-hydroxyisoleucine made use of transcriptional regulation by Lrp_Cg_ to control expression of an l-isoleucine dioxygenase gene in dependence of the l-isoleucine concentration [48]. This concept may potentially help to improve isobutylamine production. Unlike charged amino acids, such as l-glutamate or l-lysine, l-valine may passage the cytoplasmic membrane by diffusion [22, 56]. Overexpression of *brnQ*_*Cg*_ [49] improved reuptake of l-valine in a similar manner as shown previously for  l-isoleucine [54], and consequently, higher isobutylamine titers resulted. In future work, export and uptake systems for isobutylamine may be identified and used for transport engineering of the product isobutylamine in addition to transport engineering of l-valine as immediate precursor as demonstrated here.

To avoid production based on feedstocks with competing uses as food or feed, isobutylamine production based on xylose, a pentose abundant in hemicellulosics, and on orange peel hydrolysate, a food production waste, was demonstrated. Product titers were lower with xylose than with glucose, which may be due to reduced NADPH supply by the oxidative branch of pentose phosphate pathway which is directly available only with glucose. The product titer with OPH was even lower, which may be attributed to the presence of inhibitory 5-hydroxymethylfurfural in OPH as their detoxification by reduction to the alcohol requires NADPH [21, 50]. Future process intensification must improve hydrolysate preparation upstream and fermentation titers and rates midstream without compromising downstream processing for isobutylamine purification before a sustainable isobutylamine production process based on second-generation feedstocks may reach industrial relevance.

Here, isobutylamine was derived by decarboxylation of l-valine, a concept that may be extended and used for production of other SCPAs, such as 2-methylbutan-1-amine and 3-methylbutan-1-amine. The principal feasibility of producing the isopentylamine 3-methylbutan-1-amine was shown here. However, the product titer was low. Since growth inhibition of *C. glutamicum* increased with chain length of the SCPAs, ALE experiments to improve tolerance toward 2-methylbutan-1-amine and 3-methylbutan-1-amine may be required in addition to debottlenecking l-leucine and l-isoleucine biosynthesis pathways as well as improving VlmD_Sv_ activity toward either l-isoleucine or l-leucine as was demonstrated for l-alanine before [24]. In fact, this concept enabled production of the 2-oxo acids 2-oxoisovalerate, 2-oxo-3-methylvalerate and 2-oxo-isocaproate by *C. glutamicum* as consequence of abolishing their transamination to the respective BCAAs [8, 9, 26, 52]. Subsequent decarboxylation of the 2-oxo acids by KivD to the respective aldehydes and their reduction yielded the branched-chain alcohols isobutanol, 2-methyl-1-butanol and 3-methyl-1-butanol [5, 51] as first demonstrated for *E. coli* [3].

Heterologous expression of *pntAB*_*Ec*_ encoding the proton-translocating NAD(P)^+^ transhydrogenase (EC 7.1.1.1) was expected to increase isobutylamine production upon a membrane potential (ΔΨ) driven increase of NADPH supply; however, isobutylamine titers were drastically reduced (Table 1). We measured ΔΨ in response to extracellular isobutylamine addition and/or intracellular synthesis of isobutylamine, both in the presence/absence of PntAB_Ec_. Extracellular isobutylamine addition up to 100 mM to both, *C. glutamicum* WT and an isobutylamine-producing strain, reduced growth and increased ΔΨ. Hyperpolarization has also been described for ammonia in *E. coli* [2, 20]. Compared to ammonia, isobutylamine is expected to have a larger effect on ΔΨ hyperpolarization as the lipophilicity of SCPAs correlates positively with the alkyl chain length. A specific isobutylamine transport system is not known in *C. glutamicum*. Isobutylamine (pK_a_ of ~ 10.7) is predominantly present in its protonated form (isobutylammonium) at neutral pH. Uptake of the minor fraction of uncharged isobutylamine by diffusion followed by intracellular protonation to isobutylammonium increases the membrane potential, since an intracellular proton is consumed (Fig. 6B, C). Transport proteins of the Amt/Mep/Rh family of ammonium transport proteins mediate translocation of NH_3_, which at neutral pH implies extracellular deprotonation and intracellular reprotonation. Therefore, uniport of NH_3_ is considered a net antiport of NH_4_^+^ vs. H^+^ [2]. Assuming that isobutylammonium translocation is mediated in a similar manner as (methyl)ammonium translocation by AmtA and AmtB transport systems in *C. glutamicum* [10, 44], an antiport of isobutylammonium vs. H^+^ can be proposed (Fig. 6A, B). The identification of the isobutylammonium exporter could prove useful for future strain engineering. Given the potential of intracellular accumulation of isobutylammonium to contribute to product toxicity, the expression of a native or heterologous exporter could enhance product tolerance and increase production titers. An alternative strategy to mitigate intracellular isobutylammonium toxicity could involve the secretion of VlmD_Sv_ via the Tat or Sec pathway, as opposed to the deletion of *brnFE* or the overexpression of *brnQ*. However, as protein secretion can be constrained by the capacity of the secretion machinery, further engineering may be necessary to achieve sufficient secretion levels [23].

When isobutylamine is synthesized intracellularly via decarboxylation of l-valine by VlmD, ΔΨ increases as one cytosolic H^+^ is consumed in this reaction (Fig. 6B, D). This principle is known from the acid stress response of enteric bacteria, such as *E. coli,* where an amino acid such as l-lysine is taken up, decarboxylated and the generated amine is exported. These systems involve an amino acid decarboxylase and amino acid/amine antiporter to reduce the intracellular H^+^ concentration [28]. Here, we propose that the increase of ΔΨ by l-valine decarboxylation is balanced by a reduction of ΔΨ upon isobutylamine translocation out of the cell, as net antiport of isobutylamine vs. H^+^ [2]. Thus, as observed for *C. glutamicum* WT, an isobutylamine-producing strain showed no membrane hyperpolarization without extracellular addition of isobutylamine.

PntAB_Ec_ dissipates hyperpolarization by intracellular biosynthesis via VlmD_Sv_ regardless of whether isobutylamine is added extracellularly (Fig. 6B, E). As a consequence of depolarization of ΔΨ, ATP cannot be synthesized by F₁F₀–ATPase that depends on the transmembrane pH gradient. This drastically curtails growth and isobutylamine production. More generally, it is expected that production of other SCPAs by amino acid decarboxylation is hampered upon introduction of PntAB_Ec_.

Although the observed membrane potential phenotype is likely related to altered transhydrogenase activity and intracellular redox balance, the exact mechanism remains speculative as NADPH/NADP^+^ and NADH/NAD^+^ ratios were not directly measured. Subsequent studies that examine these redox ratios will be essential to further elucidate the underlying mechanism.

## Conclusion

*C. glutamicum* was engineered for de novo production of isobutylamine. In 3 L bioreactor cultivation operated in fed-batch mode, a *Y*_p/s_ of 0.47 mol/mol, a volumetric productivity *r*_p_ of 0.52 g/L/h and a titer of about 33 g/L isobutylamine were reached. To the best of our knowledge, this is the highest reported titer of a biotechnologically produced SCPA in minimal medium. Isobutylamine production based on either xylose or orange peel hydrolysate as second-generation feedstocks highlighted the potential for a sustainable production process based on renewables and side streams. Future work of process intensification will be required to reach maturity.

## Supplementary Information

Additional file 1.

## Data Availability

All data generated or analyzed during this study are included in this published article and its supplementary information files.
