# Supplementary material for: Metabolic engineering strategies for sustainable de novo isobutylamine production by Corynebacterium glutamicum
Source: Biotechnol Biofuels Bioprod. 2026 Oct 11;19:80. doi: 10.1186/s13068-026-02831-w (PMC13654226; doi:10.1186/s13068-026-02831-w)
Supplement: Supplementary file 1 [file 13068_2026_2831_MOESM1_ESM.pdf]

Supplementary Material to

Metabolic engineering strategies for sustainable *de novo* isobutylamine production by *Corynebacterium glutamicum*

Tim PRASUN[1], Susanne MÜLLER[2], Fynn STEGELMANN[1], Bastian BLOMBACH[2,3], Volker F. WENDISCH[1*]

[1]Genetics of Prokaryotes, Faculty of Biology and Center for Biotechnology (CeBiTec), Bielefeld University, Bielefeld, Germany

[2]Microbial Biotechnology, Campus Straubing for Biotechnology and Sustainability, Technical University of Munich, Straubing, Germany

[3]SynBiofoundry@TUM, Technical University of Munich, Straubing, Germany

*** Correspondence:**

Volker F. WENDISCH

volker.wendisch@uni-bielefeld.de

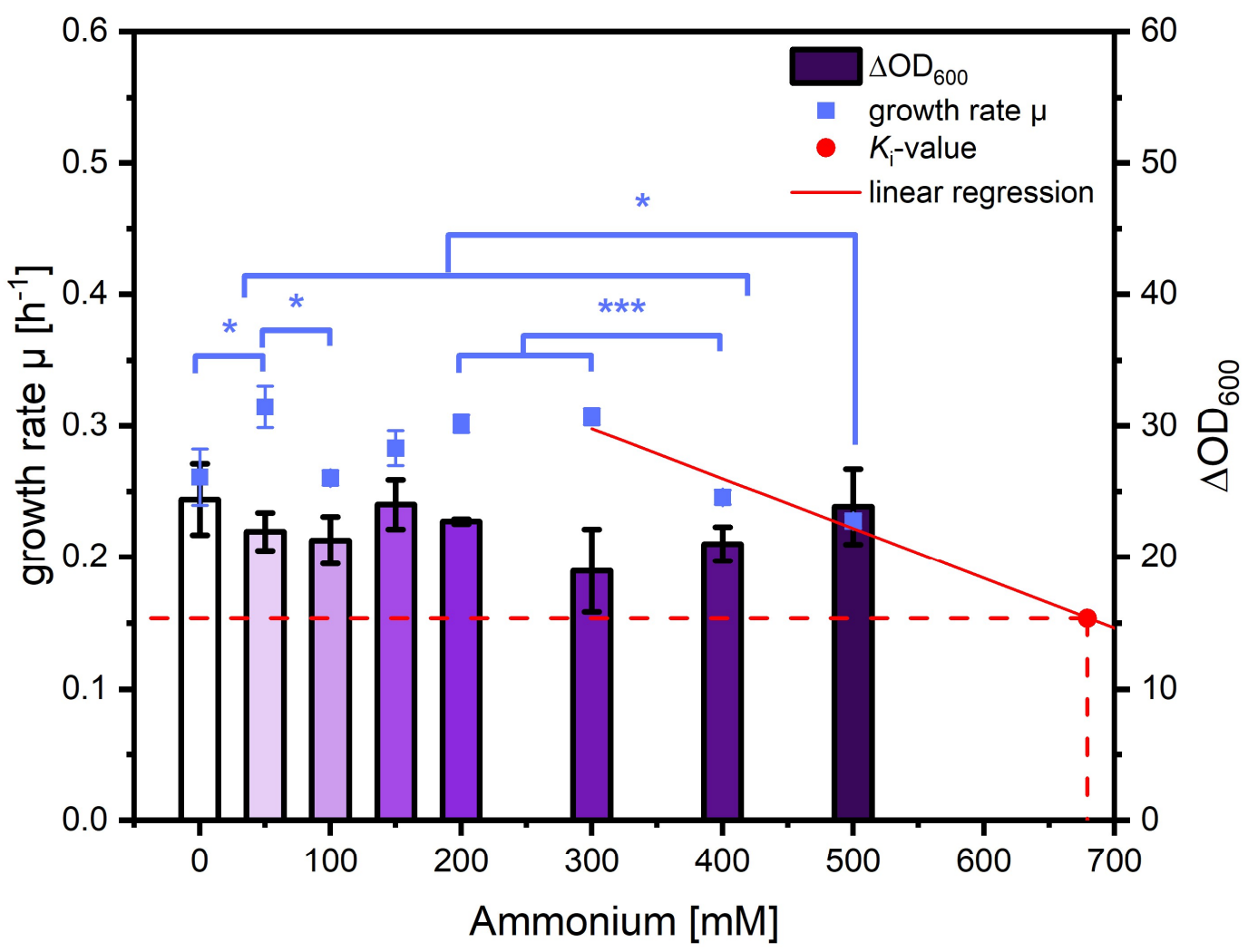

Figure S1: The growth of *C. glutamicum* WT on different ammonium (C0) concentrations. Depicted are the growth rate μ (blue squares), ΔOD_{600} (columns) and the K_i value (red dot) derived from the linear regression (red line). Darker colors indicate higher concentrations. Cultivations were conducted in culture triplicates (n=3).

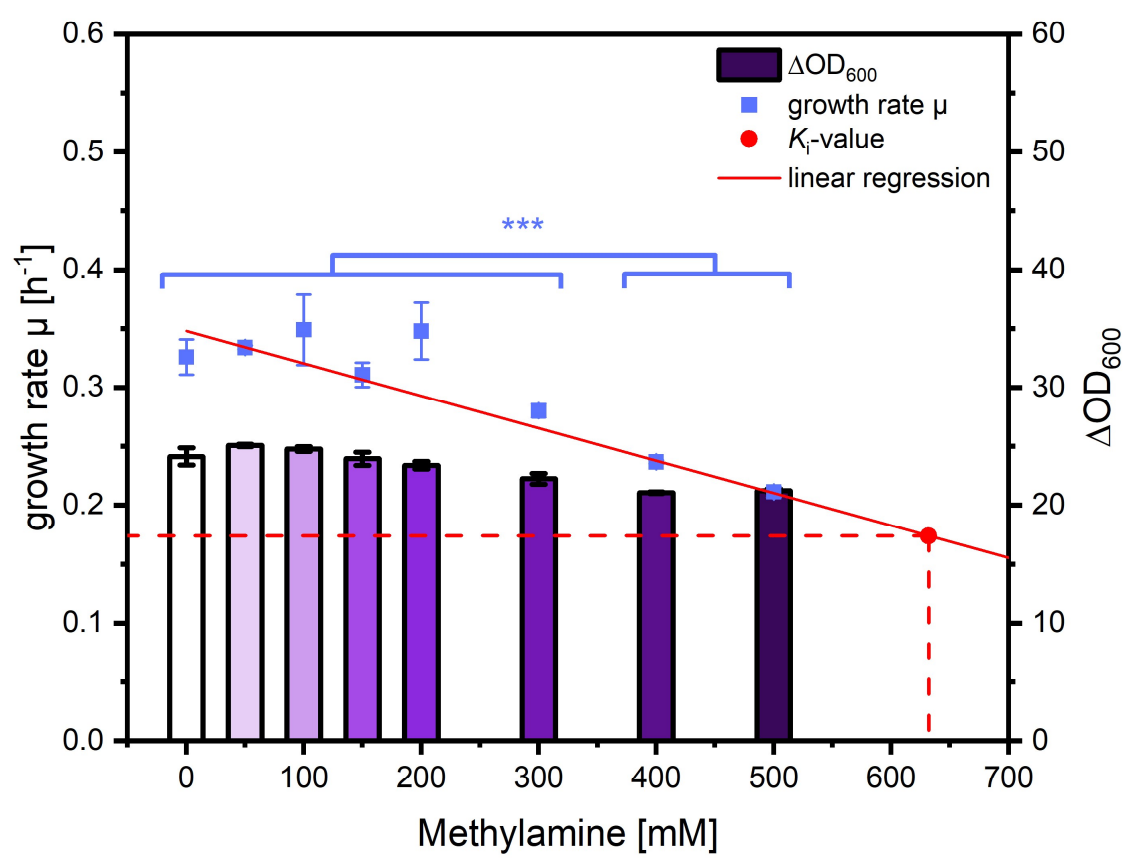

Figure S2: The growth of *C. glutamicum* WT on different methylamine (C1) concentrations. Depicted are the growth rate μ (blue squares), ΔOD_{600} (columns) and the K_i value (red dot) derived from the linear regression (red line). Darker colors indicate higher concentrations. Cultivations were conducted in culture triplicates (n=3).

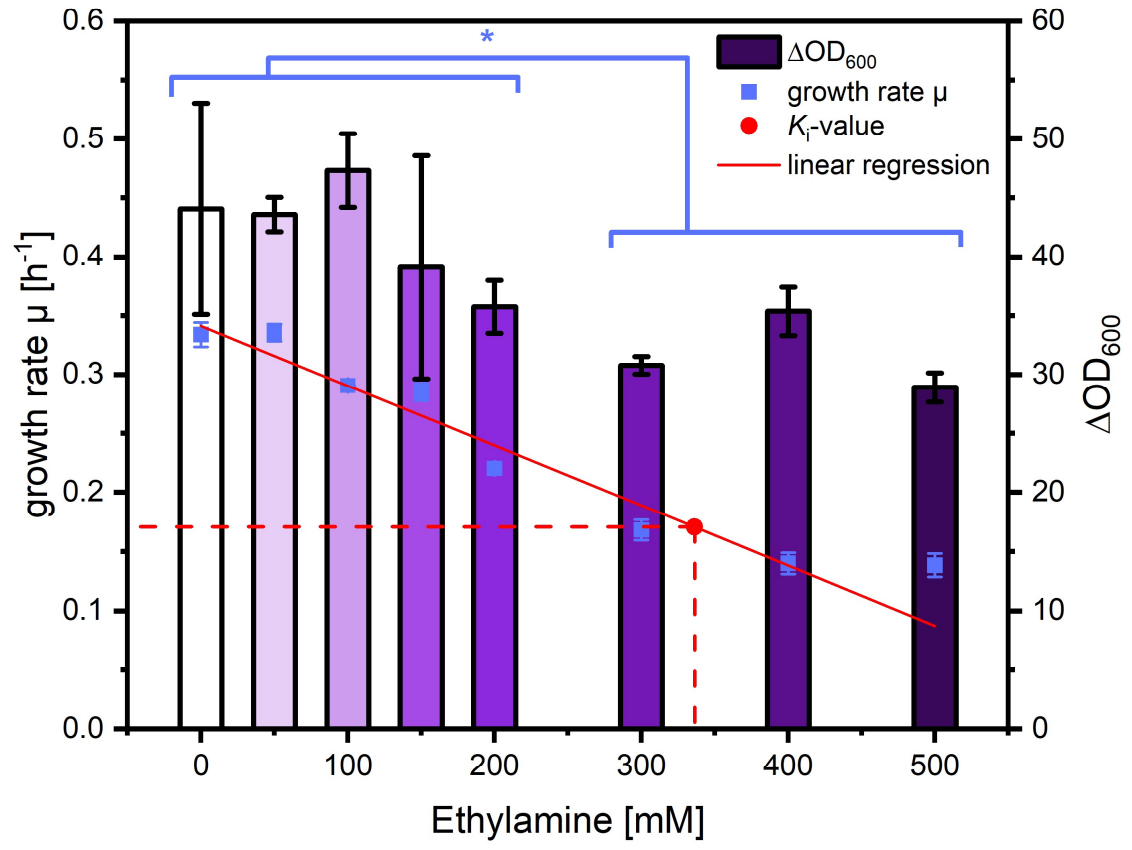

Figure S3: The growth of *C. glutamicum* WT on different ethylamine (C2) concentrations. Depicted are the growth rate μ (blue squares), ΔOD_{600} (columns) and the K_i value (red dot) derived from the linear regression (red line). Darker colors indicate higher concentrations. Cultivations were conducted in culture triplicates (n=3).

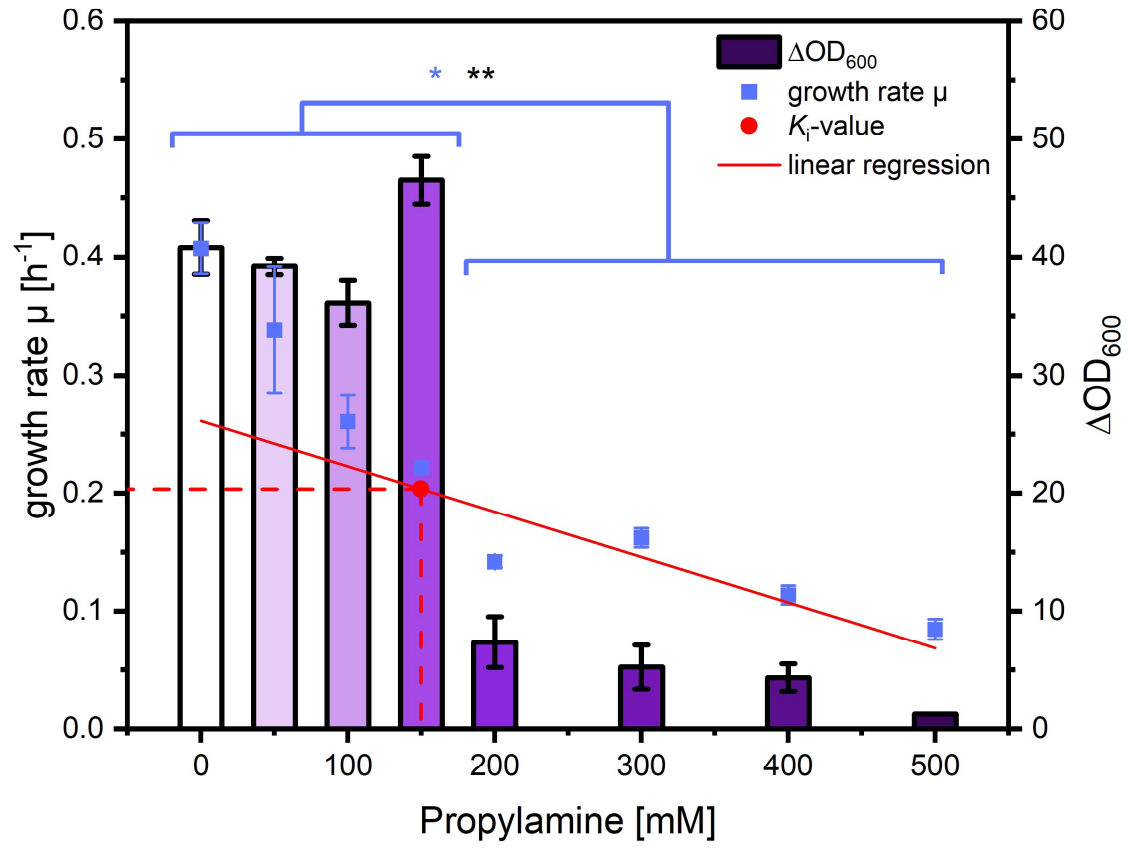

Figure S4: The growth of *C. glutamicum* WT on different propylamine (C3) concentrations. Depicted are the growth rate μ (blue squares), ΔOD_{600} (columns) and the K_i value (red dot) derived from the linear regression (red line). Darker colors indicate higher concentrations. Cultivations were conducted in culture triplicates (n=3).

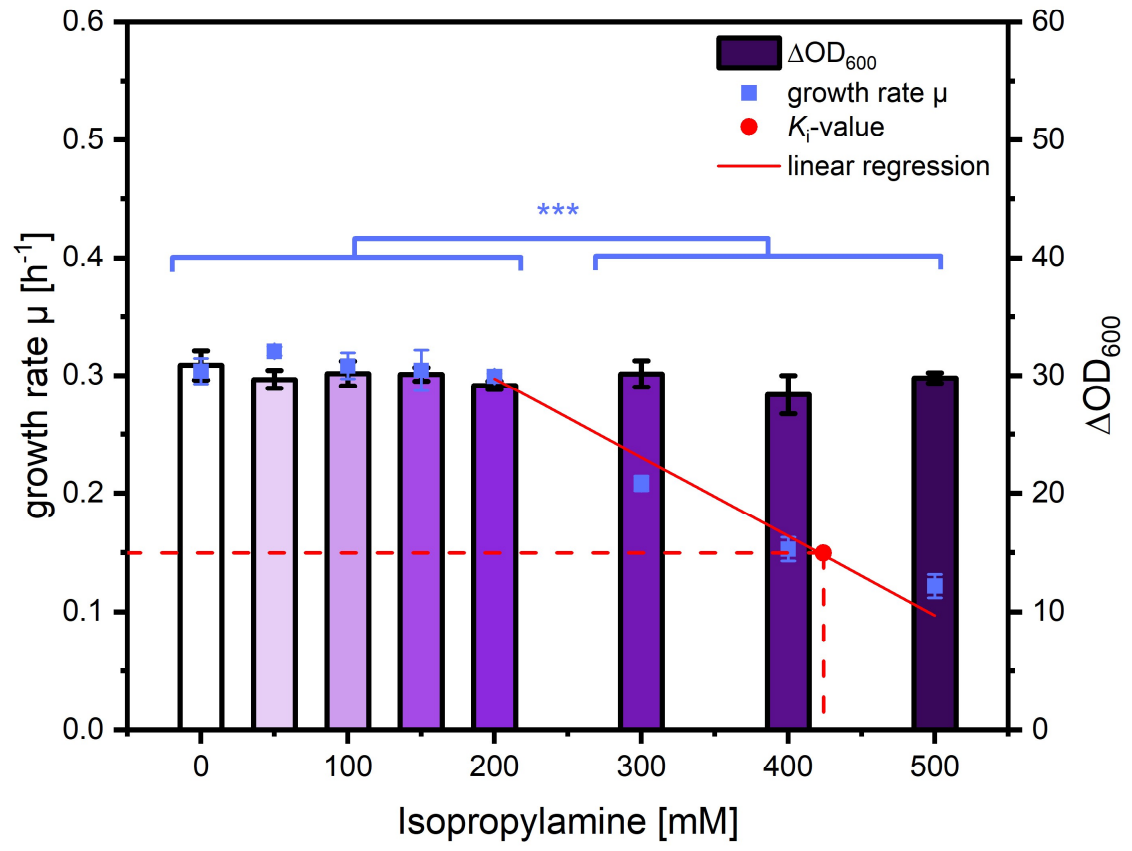

Figure S5: The growth of *C. glutamicum* WT on different isopropylamine (C3) concentrations. Depicted are the growth rate μ (blue squares), ΔOD_{600} (columns) and the K_i value (red dot) derived from the linear regression (red line). Darker colors indicate higher concentrations. Cultivations were conducted in culture triplicates (n=3).

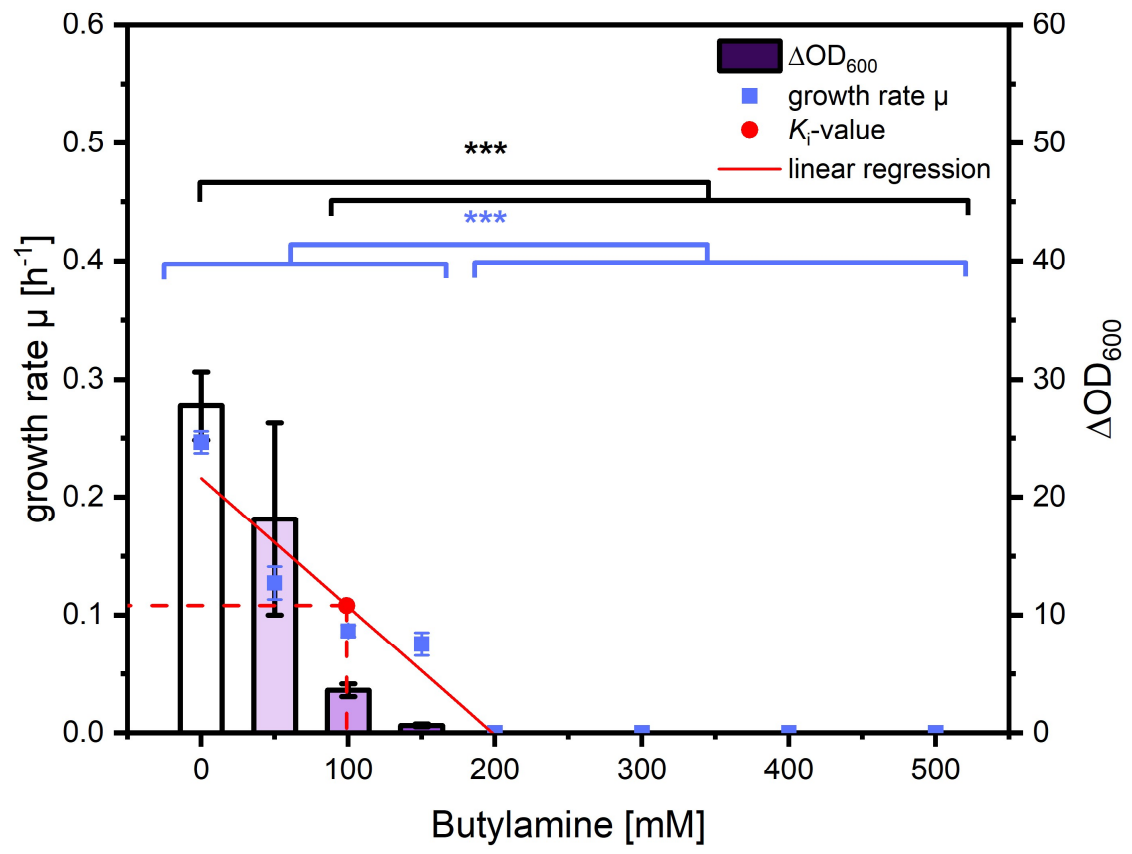

Figure S6: The growth of *C. glutamicum* WT on different butylamine (C4) concentrations. Depicted are the growth rate μ (blue squares), ΔOD_{600} (columns) and the K_i value (red dot) derived from the linear regression (red line). Darker colors indicate higher concentrations. Cultivations were conducted in culture triplicates (n=3).

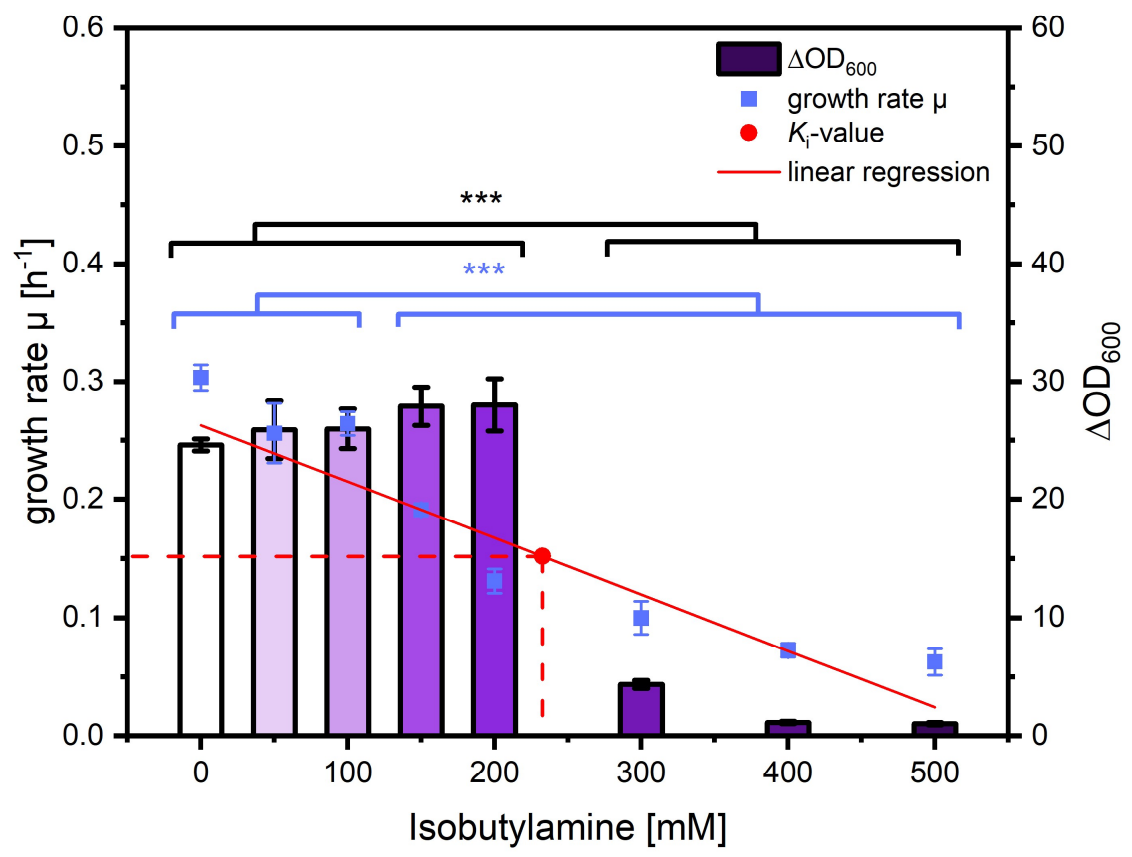

Figure S7: The growth of *C. glutamicum* WT on different isobutylamine (C4) concentrations. Depicted are the growth rate µ (blue squares), ΔOD_{600} (columns) and the K_i value (red dot) derived from the linear regression (red line). Darker colors indicate higher concentrations. Cultivations were conducted in culture triplicates (n=3).

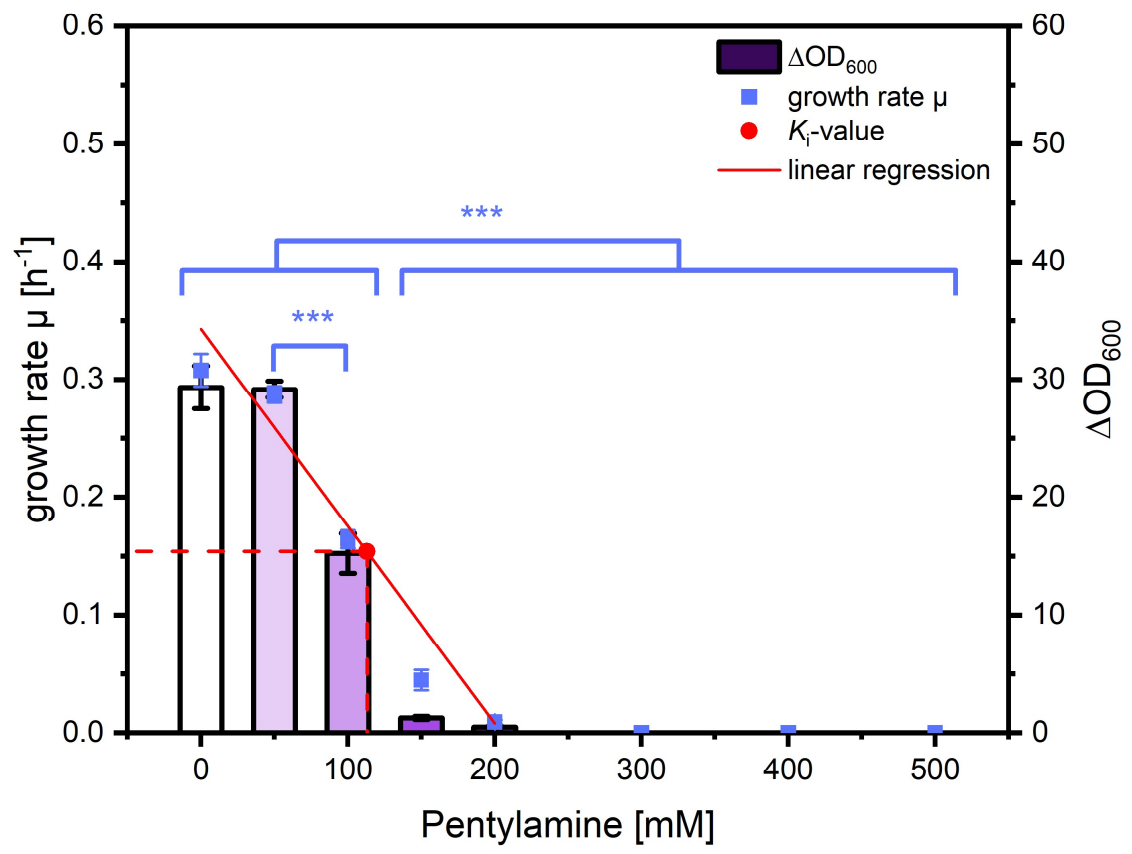

Figure S8: The growth of *C. glutamicum* WT on different pentylamine (C5) concentrations. Depicted are the growth rate µ (blue squares), ΔOD_{600} (columns) and the K_i value (red dot) derived from the linear regression (red line). Darker colors indicate higher concentrations. Cultivations were conducted in culture triplicates (n=3).

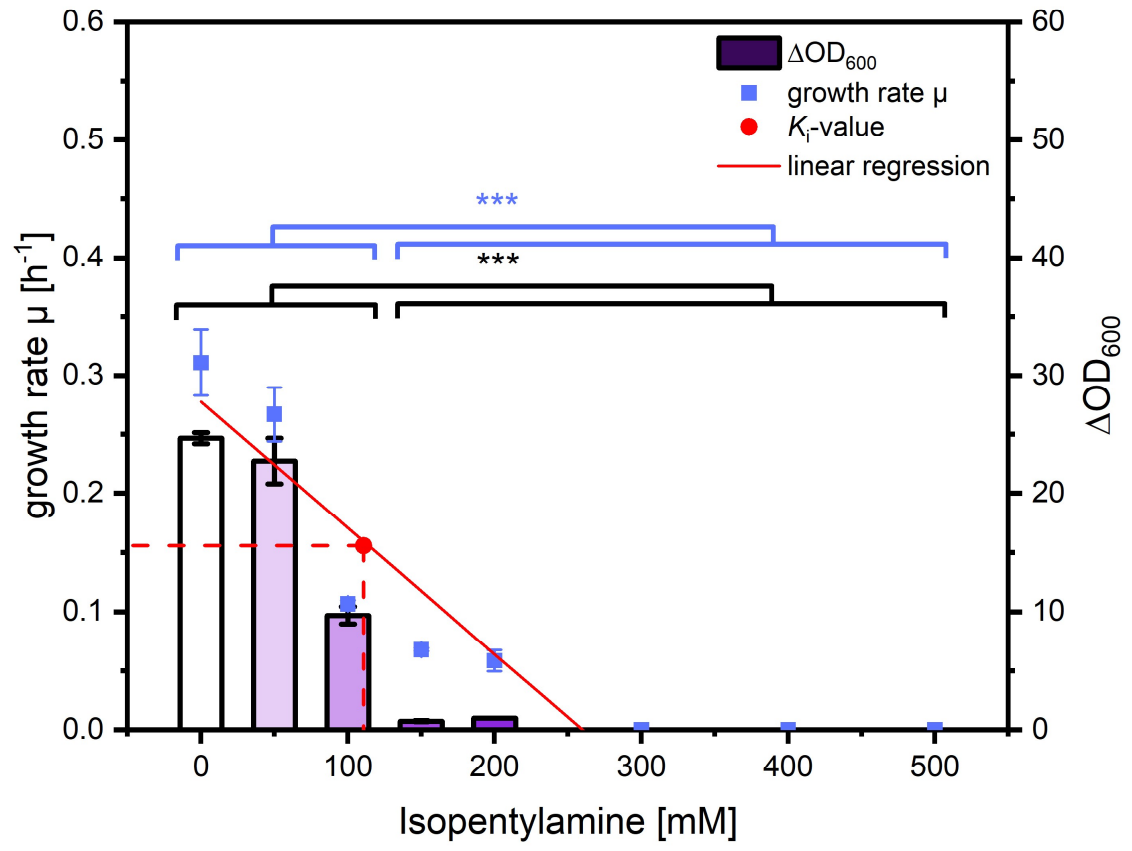

Figure S9: The growth of *C. glutamicum* WT on different isopentylamine (C5) concentrations. Depicted are the growth rate μ (blue squares), ΔOD_{600} (columns) and the K_i value (red dot) derived from the linear regression (red line). Darker colors indicate higher concentrations. Cultivations were conducted in culture triplicates (n=3).

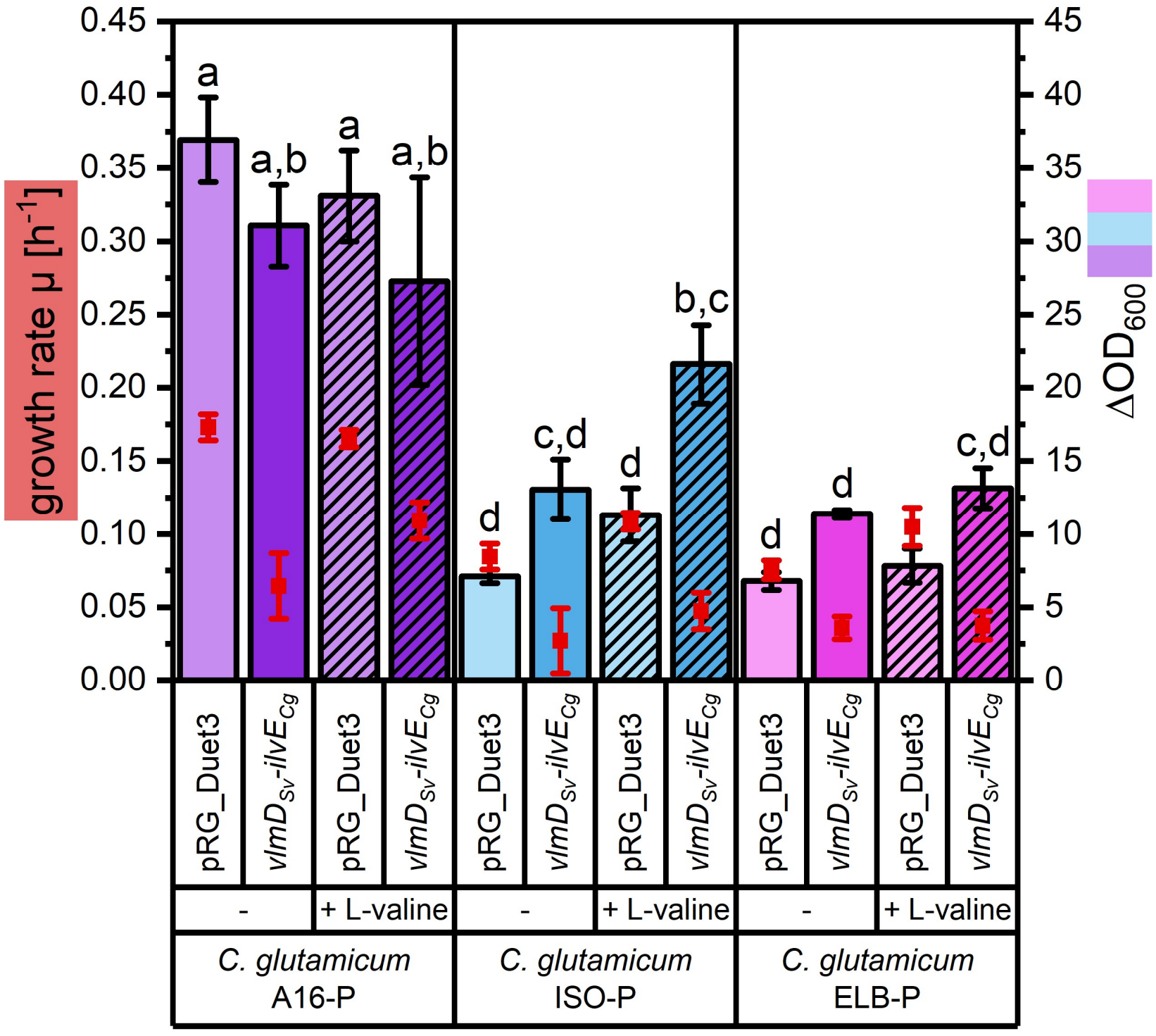

Figure S10: The growth of isobutylamine producing *C. glutamicum* strains. Depicted are the growth rate μ (red squares), ΔOD_{600} (columns). Black stripes indicate addition of L-valine. Error bars indicate standard deviation, n = 3. Supplement for Figure 3.
